# Pregnant women autonomy when choosing their method of childbirth: Scoping review

**DOI:** 10.1371/journal.pone.0304955

**Published:** 2024-07-11

**Authors:** Carlos Henrique Mascarenhas Silva, Cláudia Lourdes Soares Laranjeira, Wallisson Fonseca Pinheiro, Carolina Soares Barros de Melo, Vitor de Oliveira Campos e Silva, Augusto Henrique Fulgêncio Brandão, Francisca Rego, Rui Nunes

**Affiliations:** 1 Faculty of Medicine of the University of Porto, Porto, Portugal; 2 Obstetrics and Gynecology Unit - Mater Dei Health Network, Belo Horizonte, Brazil; 3 School of Medical Sciences of Minas Gerais, Belo Horizonte, Brazil; 4 Federal University of Minas Gerais, Belo Horizonte, Brazil; United Arab Emirates University, UNITED ARAB EMIRATES

## Abstract

This paper has as its theme the autonomy of pregnant women in relation to choosing the method of birth for their child. The objective was to carry out a scoping review to study the literature and evidence of how autonomy is being offered to parturient women. Study design and location: In October 2023, a search was carried out using the terms "pregnant women" AND "delivery" AND "autonomy" in the following databases: PubMed, Web of Science, Scopus, Scielo and LILACS. The search included articles from 2016 to 2023. Of the 179 articles found, 15 met the criteria and were selected for this review. Results: the pregnant woman’s autonomy in choosing the method of childbirth is influenced by several factors, such as the obstetrician’s recommendation, the medical team, and negative and positive experiences. Thus, when this autonomy is shared with the obstetrician, the obstetrician recommends cesarean section as the safest route, but does not explain the benefits and harms of both routes (vaginal and cesarean section), causing the woman to accept the cesarean section. Midwives recommend vaginal birth because they believe it to be natural and safe and explain the benefits and harms of both methods, respecting the pregnant woman’s choice of the method she prefers. Conclusion: women have the fundamental right to choose their method of birth and must be properly guided throughout prenatal care, whether by an obstetrician or a midwife, about the options, risks and benefits of each method of childbirth, respecting the ethical principle of beneficence.

## Introduction

Obstetric care has undergone important and impactful transformations since the beginning of this millennium, changes have notably occurred in the care model provided and questions arise to traditional medical practices that were already so consolidated and structured. Essentially, there was a transformation in the perception of childbirth, which began to be seen not only as a biological process, but as a phenomenon that involves social, cultural and emotional aspects, with greater openness to discuss and share experiences related to childbirth, breaking with the traditional, more reserved view of this moment. Thus, this holistic approach to childbirth has brought with it the attention of several professions, including nursing, physiotherapists and midwives [1].

The four principles of bioethics, which are autonomy, justice, beneficence and non-maleficence, are directly related to medical practice and specifically to labor and birth [2]. Therefore, there are multiple factors and perspectives that may be relevant to an ethical analysis using these principles. There are multiple stakeholders involved in assisting the pregnancy-puerperal cycle, and the concepts of bioethics will be presented in different forms of application and interpretation about each one of them, because, in addition to the woman herself, we also have the interests of the fetus and the values and concepts of professionals involved in clinical care practice [1].

Pregnant women and physicians do not have the same perception and the same values about the moment of giving birth or the manner of childbirth. Therefore, it is essential that health professionals are always able to offer individualized guidance, based on updated information and in line with the scientific evidence, when discussing, with pregnant women, the options for the method of childbirth, which could be vaginal or caesarean birth [3, 4]. Vaginal birth itself can currently have different care scenarios with greater or less intervention, such as the type of pain relief method (pharmacological and/or non-pharmacological), the use or not of vacuum extractor and forceps, positions of birth, place of birth (in or out-of-hospital). Therefore, it is important for the care team involved to offer knowledge about the two methods of childbirth, as a basis for the woman’s decision making [3, 4], refraining from opinions formulated based on personal experiences and beliefs.

In childbirth, we are dealing with a natural and everyday event and not with pathological conditions, but one must be aware that, despite being a physiological process, it has, on the other hand, great potential to become acutely serious and urgent. Furthermore, the entire process of pregnancy and childbirth has a great impact on the lives of mothers and their fetuses/children, which is often hidden during medical-hospital care [5, 6].

Considering the importance of the birth process in women’s lives and on the biopsychosocial impacts that the decisions inherent to this process have on their lives, it is expected that they want to participate in decision-making. Above all, on which method of childbirth to choose; vaginal or caesarean section [3, 4].

The decision about the method of childbirth can be influenced by several factors, such as professional considerations, women’s negative and positive experiences, health outcomes, financial issues, prolonged hospital stay and clinical complications. Some reasons that contribute to opting for a cesarean section include the desire to avoid pain, the lack of essential information or difficulties in understanding concepts related to the method of birth, the perception of lower risk associated with this procedure and the possibility of planning the moment of birth [7]. On the other hand, the preference for natural birth is based on the prospect of a faster recovery, less intensity of postpartum pain and the opportunity for women to play a central role during the childbirth process [8, 9].

Therefore, the pregnant woman’s autonomy in choosing the method of childbirth via cesarean or vaginal childbirth must be respected and supported by medical societies in their respective jurisdictions.

The American College of Obstetricians and Gynecologists (ACOG), for example, recommends that pregnant women share decision-making with their doctor, based on US care protocols [10]. The Royal College of Obstetricians and Gynaecologists (RCOG) in the United Kingdom recommends that health care teams support the mother’s request for a cesarean section if the woman is sure that she wants it [11]; this also reflects clinical guidance from the National Institute for Health and Care Excellence (NICE). They do not require that it be a decision shared with a doctor, the doctor is expected to support the woman’s choice, even if the doctor does not share the belief that it is the correct choice [12].

That said, by debating and promoting the autonomy of pregnant women, we contribute to the construction of a culture of respect for reproductive rights, strengthening the bond between health professionals and pregnant women. Therefore, the present study aims to evaluate the stage of discussion in current literature about the autonomy that pregnant women have to choose and have their decision to choose the method of birth for their children respected.

## Materials and methods

### Research strategy

A Scoping Review was carried out, as the researchers’ objective was to identify the knowledge gaps existing in our currently published literature about the autonomy of pregnant women in choosing their method of birth during the birth of their children. This type of study is the most appropriate for this purpose [13, 14], considering that this type of review is indicated when some topic in the literature, in this case the autonomy in choosing the method of birth, has not yet been comprehensively reviewed, or exhibits a complex nature not amenable to a precise systematic review [14].

The Scoping Review, like the systematic review, is a form of knowledge synthesis that incorporates a series of studies to summarize evidence, very useful in making practical decisions. But by dealing with themes that still have bibliographical gaps, the objective of information and guidance for the development of priorities in future research, in order to explore intensely the theme in question [15]. This study followed the Preferred Reporting Items for Systematic Reviews and Meta-Analyses for Scoping Reviews (PRISMA-ScR) guidelines.

Thus, on October 1st, 2023, an extensive bibliographical search of the available literature began, using the descriptor terms “pregnant women” AND “delivery” AND “autonomy” in the following databases: PubMed, Web of Science, Scopus, Scielo and LILACS. The research was completed on November 1st, 2023.

### Selection criteria

The inclusion criteria used were: articles published in the period comprised of the last 7 years (2016 to 2023), which evaluated the autonomy of women in childbirth care. We evaluated the women’s perception of choosing the method of birth they wanted to have and the effective autonomy of these women in decision-making. Quantitative and qualitative studies were included. It is worth noting that, during the selection, autonomy was limited to the method of birth, but autonomy in relation to satisfaction with the childbirth and assistance received was also taken into consideration.

The exclusion criteria used were: articles based on the language in which they were published were not excluded. Articles classified as case reports, newspaper news, editorials and opinion articles were excluded. We also excluded those studies that, despite assessing women’s autonomy, analyzed aspects other than the objective of this article, which is the choice of method of birth.

### Data collection

The collection and research of the articles, and the analysis of the inclusion and exclusion criteria were carried out by two researchers separately, with manual extraction of data, through reading and analyzing the abstracts of the articles. The definition of the articles to be included was made through consensus among the researchers. The initially selected articles were later submitted to another analysis, in detail, through the complete reading of the publication.

## Results

**Fig 1:**
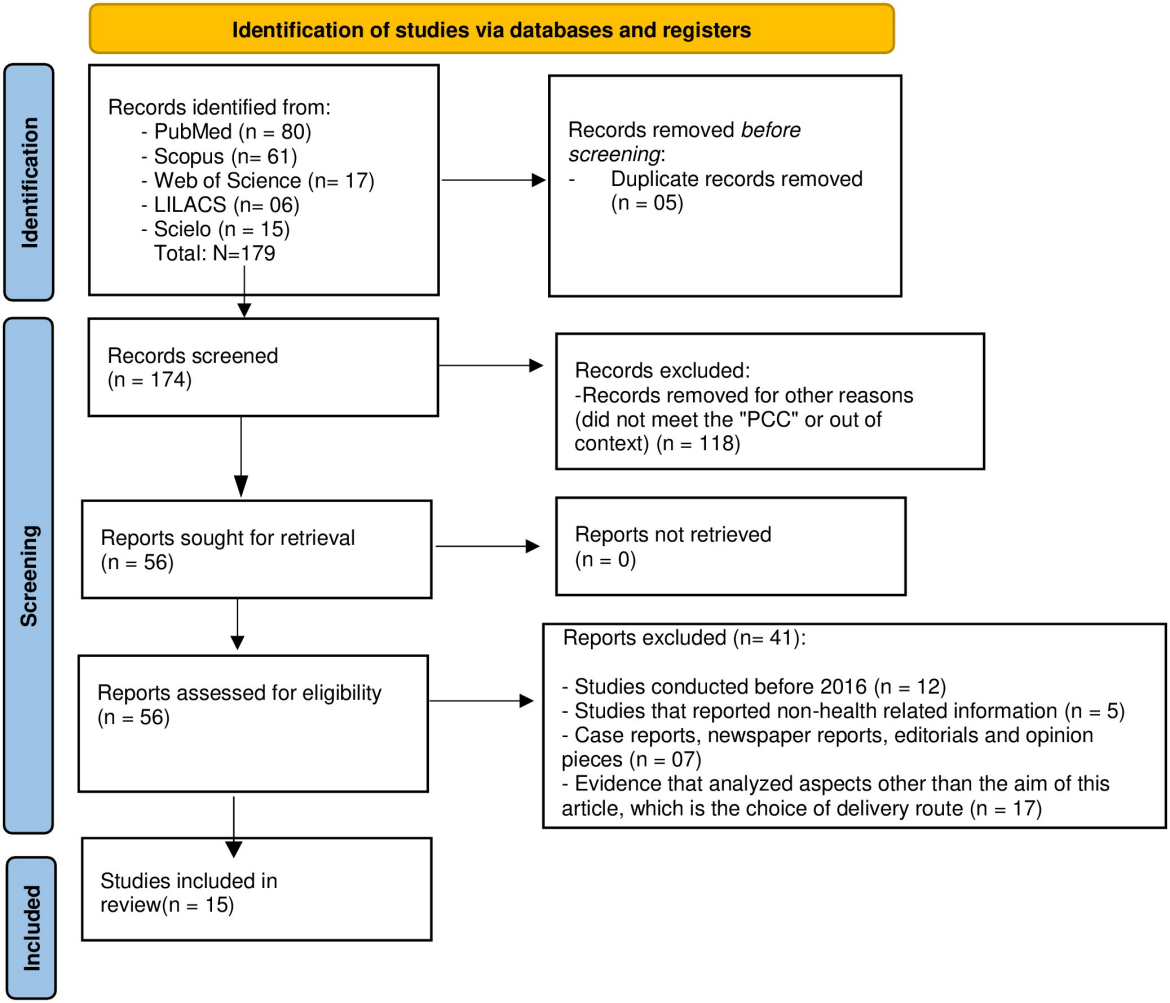
PRISMA 2020 flow diagram for new systematic reviews which included searches of databases and registers only.

The research found 179 articles in the 5 cited databases, being found in the databases 80 articles in PubMed, 17 in Web of Science, 61 in Scopus, 15 in Scielo, 06 in LILACS, and 05 of these works were duplicated and were excluded. Afterward, the titles and abstracts of the pre-selected articles were read, excluding another 118 articles that were not directly related to the theme proposed for this review. Then, the 56 selected articles were read in full and categorized by the two reviewers. They went through the inclusion and exclusion criteria, resulting in 41 publications being excluded. During the selection, it is reiterated that autonomy was limited to the method of birth, but autonomy was also taken into consideration in relation to satisfaction with the birth and assistance received, since this satisfaction is directly related to the autonomy of the method of birth. Thus, the research resulted in 15 articles that were selected and discussed in this review. The article selection process is presented in the PRISMA flow diagram [16] for the inclusion and exclusion criteria (Fig 1).

A total of 15 articles were included in this review. It is observed that 4 (26.6%) of them are classified as literature reviews [17–20], 4 (26.6%) as intervention studies [21–24], 4 (26.6%) are prospective observational studies [25–28] and 3 (20%) are cross-sectional studies [29–31]. Furthermore, by having a parallel between the studies, we noticed that only 11 (73.3%) studies directly investigated the autonomy of women in decisions regarding the method of childbirth [17, 19–22, 24–28, 30]. Regarding the remaining studies, 3 (20%) focused on the experience and satisfaction with the care received in the maternity ward [18, 23, 29], and only 1 (6.6%) on the women’s profile and decisions [31] (Table 1).

**Table 1:** List of selected articles.

Author and year	Name of the article	Location of the study	Method	Objective	Conclusion
Nguyen (2017)	The importance of clinically and ethically fine-tuning decision-making about cesarean delivery	New York, United States of America	Intervention with the preparation of an ethical clinical document.	Develop a clinical ethical framework with clinical and organizational factors for decision-making and advice on cesarean childbirth.	The adoption of a clinical ethical framework in practice to increase the autonomy of pregnant women by providing counseling to improve their autonomy in choosing the method of childbirth.
Vedam (2017)	The Mother’s Autonomy in Decision Making (MADM) scale: Patient-led development and psychometric testing of a new instrument to evaluate experience of maternity care	British Columbia, Canada	Intervention with application of the MADM scale with 2051 women	Develop and validate a new instrument that assesses women’s autonomy and role in decision-making during maternity care.	The Mothers’ Autonomy in Decision Making (MADM) scale proves to be reliable and valid for the decision-making process in maternity care.
Vedam (2017)	The Mothers on Respect (MOR) index: measuring quality, safety, and human rights in childbirth	British Columbia, Canada	Intervention with application of the MOR index with 1,672 women	Develop a survey tool that assesses women’s experiences with maternity care, including their autonomy.	The MOR index is a reliable quality and safety indicator that can be applied across jurisdictions to assess access to autonomy-respecting maternity care.
Reis (2017)	Female autonomy in the labor and birth process birth process: an integrative literature review	Rio Grande do Sul, Brazil	Integrative literature review. With article selection criteria	Identify the evidence available in scientific production about health care practices that interfere with the exercise of autonomy by Brazilian women in the labor and childbirth process.	There is a setback in the recognition and realization of women’s rights in their fullness, making it impossible to exercise their autonomy in relation to their own bodies and childbirth.
Bohren (2017)	Continuous support for women during childbirth	Geneva, Switzerland	Systematic literature review. With article selection criteria	Evaluate the effects of continuous and individual support during childbirth	Continued support during labor can improve outcomes for women, including increased spontaneous vaginal birth, shorter duration of labor, and decreased cesarean birth.
Vedam (2019)	Patient-led decision making: Measuring autonomy and respect in Canadian maternity care	British Columbia, Canada	Intervention with application of the MADM and MOR scales exploring new topics with 2051 women	Explore women’s preferences and role in decision-making related to maternity care	Women’s autonomy is significantly altered by the maternity care model, the nature of interactions with caregivers, and women’s capacity for self-determination.
Fernandes (2019)	Profile of high-risk pregnant women and co-management of the decision on the method of childbirth between doctor and pregnant woman	São Paulo, Brazil	Cross-sectional, prospective study. With a questionnaire applied to 405 pregnant women.	Characterize the profile of high-risk pregnant women when deciding on the method of childbirth.	When women choose their birth individually, the majority choose a natural birth. When only the doctor decides, he recommends a cesarean section. And when it is decided together, a cesarean section prevails.
Loke (2019)	Is it the decision of women to choose a cesarean section as the mode of birth? A review of literature on the views of stakeholders	Hong Kong, China	Systematic literature review. With article selection criteria	To explore the decision of women with low-risk pregnancies to undergo a cesarean section as a method of childbirth.	The decision-making process about the method of childbirth is not simple and although the woman has autonomy, this can be influenced by the obstetrician, family and friends.
Feijen-de Jong (2020)	Measuring respect and autonomy in Dutch maternity care: Applicability of two measures	Groningen, The Netherlands	Intervention with application of the MADM and MOR scales with 557 pregnant women	Assess the applicability of Canadian measures; the Mothers’ Autonomy in Decision Making (MADM) scale and the Mothers Over Respect (MOR) index measures among pregnant women in the Netherlands.	They support the feasibility, reliability and validity of applying the MOR and MADM scales to assess women’s autonomy.
Tajuddin (2020)	Why women chose unassisted home birth in Malaysia: a qualitative study	Kuala Lumpur, Malaysia	Prospective observational study using a questionnaire with 12 pregnant women	Exploring women’s autonomy in relation to childbirth in Malaysia.	Women in Malaysia have complete autonomy to opt for a natural home childbirth, being able to express their personal opinions and values, to the detriment of health risks.
Schantz (2020)	Moving beyond the ethical tension of caesarean section on maternal request	Paris, France	Prospective observational study using a questionnaire with 37 women (pregnant women and midwives)	Assess whether it is ethically acceptable for midwives to accompany a woman in her decision to have a cesarean section.	Most women and midwives share a view of childbirth as something “natural”, preferring vaginal birth. This choice incorporates the ethical principles of beneficence and non-maleficence. On the other hand, midwives express the desire to respect the pregnant woman’s choice and freedom, illustrating the ethical principle of respect for autonomy.
Miller (2022)	African American Women’s Experiences with Birth After a Prior Cesarean Section	North Carolina, United States of America	Prospective observational study using a questionnaire with 25 pregnant women	Characterize the pregnancy experience and decision about method of childbirth in African-American women with a previous cesarean section.	It encourages shared decision-making between pregnant women and the medical team.
Zewude (2022)	The Preferences of Modes of Child Delivery and Associated Factors Among Pregnant Women in Southern Ethiopia	Sodo, Ethiopia	Cross-sectional survey research study, using a questionnaire with 398 pregnant women	Identify the choice of method of childbirth and the factors associated with this decision.	Most women prefer vaginal childbirth and this decision is mainly based on the expectation that this method is the most natural and the belief that it is better for the mother-baby binomial.
Sorrentino (2022)	Caesarean Section on Maternal Request-Ethical and Juridic Issues: A Narrative Review	Palermo, Italy	Systematic literature review. With article selection criteria	Describe the reasons for the growing demand for cesarean sections chosen by pregnant women	The pregnant woman’s autonomy is influenced by negative experiences that lead women to opt for surgical childbirth because they fear that the fetus will be harmed or because they want a cesarean section for cultural reasons or fear of the unknown.
Stoliar (2023)	A national survey of Australian midwives’ birth choices and outcomes	Sydney, Austrália	Prospective observational study using a questionnaire with 447 midwives	Explore the importance of midwives about their experience of giving birth in choosing the method of childbirth.	Women who are midwives generally have high rates of normal vaginal childbirths and low rates of interventions such as cesarean sections.

The year with the most publications was 2017 (4); the years 2019 (3), 2020 (3) and 2022 (3) had the same number of publications. The years 2016 and 2023 had only 1 publication each on the topic. Regarding the study location, these were selected from various jurisdictions such as Canada [22–24]; United States of America [21, 25]; Brazil [17, 31]; Switzerland [18]; China [19]; Netherlands [29]; Ethiopia [30]; Malaysia [26]; France [27]; Italy [20]; Australia [28] (Table 1). Conducting this survey of the study site is very relevant, as the way in which autonomy is supported (or not) may vary according to legal requirements around consent in different jurisdictions and also prevailing obstetric requirements.

In the articles analyzed, 4 (26.6%) of them [17–20] review the literature on the autonomy of pregnant women in choices related to childbirth; 3 (20%) apply questionnaires to women to evaluate pregnant women’s choice for the method of childbirth [26, 30, 31]; 1 (6.6%) proposes assistance intervention measures to allow pregnant women to exercise their right to choose [21]; 4 (26.6%) seek to analyze an evaluation questionnaire on the care offered during childbirth [22–24, 29]; 1 (6.6%) interviewed women who underwent a new birth after a previous cesarean section, analyzing autonomy regarding the choice of method of childbirth, experience and the influence of racism on the attitude of the professional who provided childbirth assistance [25]; and 2 (13.3%) explored the importance of midwives about their experience of giving birth in choosing the method of childbirth [27, 28] (Table 1).

**Fig 2:**
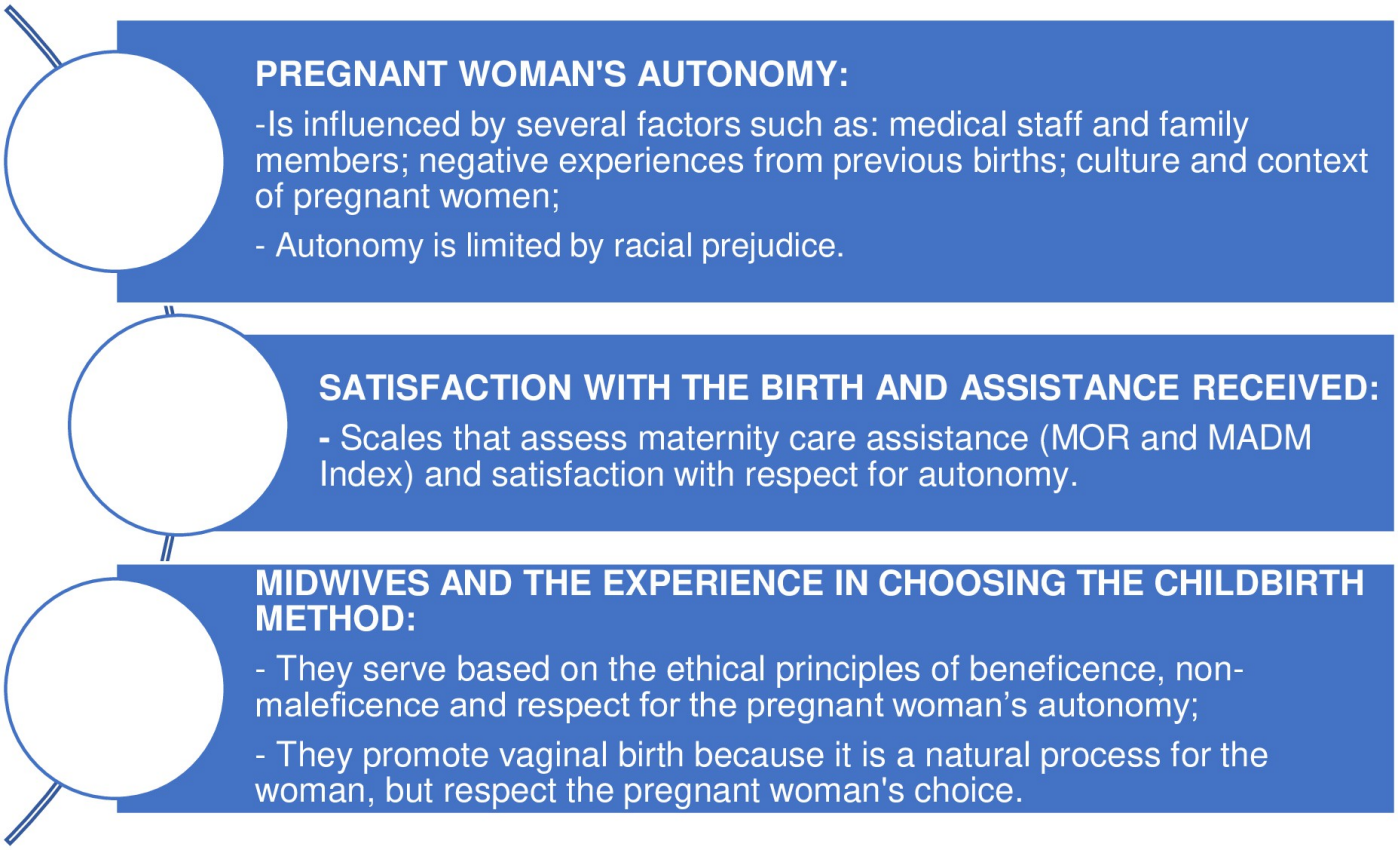
Concepts related to the analysis of the pregnant woman’s autonomy regarding the choice of childbirth method.

The analysis of the studies included in this review allowed the identification of the following outcomes associated with the topic addressed, called: women’s autonomy; satisfaction with the birth and assistance received; midwives and their experience in choosing the method of childbirth (Fig 2).

Women’s autonomy: The impact of autonomy on pregnant women’s choices is an interesting point to be evaluated. It was evidenced by the author Loke that the decision-making process about the method of childbirth is not simple and although the woman has autonomy in choosing, this can be influenced by the obstetrician and medical team, as well as by family and friends [19]. Thus, the author Vedam, in two large studies carried out in Canada, developed intervention measures aimed at helping women have autonomy in decision-making during maternity care [22, 24]. Thus, the Mothers’ Autonomy in Decision Making (MADM) scale was developed, an instrument that demonstrates that it is reliable and valid for the decision-making process in maternity care. It is important to highlight that here the author lists the autonomy of care procedures in the maternity ward and does not mention the methods of childbirth [22]. Vedam, in a study two years later, concluded that women’s autonomy is significantly altered by the maternity care model, the nature of interactions with caregivers, and women’s capacity for self-determination [24].

Reis states that there is a setback in the recognition and realization of women’s rights in their totality, making it impossible for pregnant women to exercise their autonomy in relation to their own bodies and childbirth [17]. Miller, in their article on African-American women’s experiences with childbirth, highlighted the presence of limited autonomy, lack of support, and negative experiences related to racial bias with providers [25].

The first author to mention the methods of childbirth was Nguyen, who, in a study carried out in the USA, proposed the adoption of a clinical ethical framework to guide the obstetrician and assist in the shared autonomy of choosing the method of childbirth, suggesting an evidence-based analysis in relation to the choice of cesarean section [21]. Fernandes states that when women choose their method of childbirth individually, the majority of them choose natural birth. But when only the doctor decides, the doctor recommends a cesarean section, and when a joint decision is made, the cesarean section prevails [31]. Sorrentino highlights that the pregnant woman’s autonomy is influenced by negative experiences that lead women to opt for surgical childbirth because they fear that the fetus will be harmed or because they want a cesarean section for cultural reasons or fear of the unknown [20].

On the other hand, Zewude states that the majority of women prefer the vaginal childbirth method, and this decision is mainly based on the expectation that this method is the most natural and the belief that it is better for the mother-baby bond [30]. Tajuddin points out that women in Malaysia have complete autonomy to choose natural or vaginal birth, the latter at home, being able to express their opinions and personal values, to the detriment of their health [26].

Satisfaction with the birth and assistance received: several studies have demonstrated satisfaction with childbirth and the assistance received, showing that the implementation of the right to choose the method of childbirth, and the perception of this autonomy can be guaranteed, combined with continuous and individual support, which results in increased satisfaction for pregnant women [18, 23, 29]. Vedam developed the MOR index, which is a reliable quality and safety indicator that can be applied in all jurisdictions to assess access to maternity care with respect for the autonomy of women, aiming to favor this autonomy [23]. Bohren states in her study that continued support during labor can improve outcomes for women, including increased spontaneous vaginal childbirth, shorter duration of labor and decreased cesarean section childbirths. Feijen-de Jong carried out a study adapting and applying the MADM and MOR scales in the Dutch healthcare system to evaluate the assistance and autonomy satisfaction of women, showing that satisfaction with assistance guarantees better autonomy for women [29]. Thus, Miller reiterates that decision-making, when shared between the pregnant woman and the medical team, presents greater satisfaction [25].

Midwives and experience in choosing the method of childbirth: the role of midwives in monitoring labor and childbirth is evident in some countries, such as France and Australia, reflecting on the process of building the autonomy of pregnant women [27, 28]. Schantz pointed out that midwives and women cared for by midwives share a view of childbirth as something “natural,” preferring vaginal childbirth. This choice incorporates the ethical principles of beneficence and non-maleficence. On the other hand, midwives express the desire to respect the woman’s choice and freedom, illustrating the ethical principle of respect for autonomy [27]. Stoliar concludes that women who are midwives generally have high rates of vaginal childbirths performed and low rates of interventions such as cesarean sections [28].

## Discussion

Autonomy is the ability of a conscious and capable individual to make decisions and choices, without pressure, after being properly guided [32, 33]. The autonomy of women in choosing the method of childbirth has been debated for a long time, and, when respected and promoted, contributes to a more individualized and pregnant-centered experience, reflecting an approach to care that values autonomy as a fundamental principle.

In 1979, it was established during the United Nations International Convention on the Civil and Political Rights of Women, associated with Article 1 of the Convention on the Elimination of All Forms of Discrimination against Women (CEDAW), that “any distinction, exclusion or restriction based on sex and which has the effect or purpose of diminishing or nullifying the recognition, pleasure, or exercise by women, regardless of their marital status, must be banned, based on equality between men and women, human rights and fundamental freedoms in the civil fields, politics, economy, society, culture, or any other area” [34].

In this way, guaranteeing women’s right to choose regarding their method of childbirth is in absolute agreement with the proposals contained in the aforementioned declarations. Capable individuals have the right to make their choices, when these do not harm their health, consenting to or denying medical treatment. In the scope of obstetric care, the woman must be at the center of care and have her expectations and desires met, respecting the safety limits for the health of the mother and fetus [34].

Thus, upon analysis of the results obtained, it was possible to notice that the authors report that decision-making regarding maternity care and the method of childbirth, although it is a woman’s autonomous right, is influenced by several factors such as the care model of maternity, through interactions with care providers (the team that provides care during childbirth), with family and friends, and through the negative or positive experiences of women [17, 19, 22, 24, 25].

These influencing factors are evidenced by other authors in the literature, such as Neves, who stated that autonomy in the process of choosing the method of childbirth is constructed by the influence of prenatal care, by the health sector responsible for care, by biopsychosocial aspects and knowledge of pregnant women in relation to each birth [8].

The influence of the medical team is highlighted mainly in Fernandes’ study, as when women were able to choose the method of childbirth individually, they chose natural childbirth; when they left it up to the doctor to choose, the doctor opted for a cesarean section; and when the decision was shared, the cesarean section prevailed [31].

When the influence is the result of the experiences lived by the woman, the choice of the method of childbirth will be favored depending on how negative or how positive it was, as for example in the study by Sorrentino, who pointed out that, due to negative experiences in the natural childbirth method, the women opted for cesarean section [20]. When the experiences are positive or they believe that the natural childbirth method is the most natural and healthy for both the mother and the baby, they opt for a vaginal childbirth [26, 30].

The pregnant woman, in turn, inserted in a social context, has values, feelings, beliefs and experiences that intertwine to shape her representations and decisions about the birth process. They, therefore, highlight the complexity of the decision-making process regarding method of childbirth during pregnancy [7, 9].

The institutionalization and medicalization of childbirth have transformed this moment into a pathological process, mediated by unnecessary interventions, and it is up to the professional obstetrician or midwife who will monitor maternity care to guide the pregnant woman on the most viable method of childbirth for her situation. This is consistent with the findings of Nguyen’s study, which, aiming to implement shared autonomy in choosing the method of childbirth, developed a clinical ethical framework to guide the obstetrician in the evidence-based analysis regarding the choice of cesarean section [21].

When we relate Autonomy with two other bioethical precepts, which are Beneficence and Justice, there is a discussion about the need to offer or not offer cesarean section on request only to women who request it, or whether it would be an ethical obligation of the attending physician to offer this option of childbirth for all pregnant women. It is considered here that the same opportunities and treatment options must be offered to all women, and not doing so would be a form of discrimination. Those who argue in favor of this theory understand that the offer must be associated with general guidance on all birth options, their advantages and disadvantages, with the doctor having the role of assisting and supporting the decision [33]. Others understand that this is not necessary, as women who want access to a cesarean section upon request will voluntarily make such a request [34, 35].

Daily medical practice presents countless situations where the precepts of bioethics are presented in a clear and explicit way; in others, this is not clear, and medical attention is essential so that respect for the principles is followed. Knowing and applying such principles in women’s care can help obstetricians to resolve complex ethical situations more easily, in addition to facilitating the establishment of an effective relationship between the woman and her doctor [1]. At these moments, understanding the situations peculiar to each woman’s life, their personal, family and biosocial values become essential for good medical practice, measurable through the clinical and psychological results obtained at the end of care [10].

In relation to satisfaction with childbirth and assistance received, several studies highlight that the implementation of the right to choose the method of childbirth, combined with the perception of autonomy, proves to be crucial to guarantee the satisfaction of pregnant women. This satisfaction is amplified when accompanied by continuous and individualized support, highlighting the relevance of integrating these elements for a positive experience during the childbirth process. Both satisfaction and autonomy of pregnant women can be assessed with the MADM and MOR scales [18, 23, 25, 29].

These MADM and MOR scales, developed by Vedam, respectively assess autonomy and the role of women in decision-making; and women’s experiences of respect when interacting with their healthcare professionals. Used to assess women’s autonomy, respect and overall childbirth experience, these scales are highly relevant for research purposes as well as clinical settings. The measured experiences can be used as a contribution to develop and optimize maternal care, resulting in respect for maternal care and the autonomy of the woman [36, 37].

The obstetrician and the hospital team were not the only ones identified in the studies as responsible for monitoring the maternal care of women, with the participation of midwives being evidenced in some jurisdictions. Thus, it was observed in relation to the outcome regarding midwives and the experience in choosing the method of childbirth, that this approach reveals the ethical complexity in the context of childbirth, especially when it comes to the midwives’ view [27, 28].

The preference for vaginal childbirth as something "natural" reflects the appreciation of the ethical principles of beneficence, seeking what is considered best for the health and well-being of the pregnant woman. Furthermore, by prioritizing this method, midwives can be aligned with the principle of non-maleficence, avoiding unnecessary interventions. At the same time, the midwives’ commitment to respecting women’s choice and freedom is notable, highlighting the ethical principle of respect for autonomy. This duality between the "natural" perspective and respect for autonomy highlights the delicate ethical balance faced by midwives, seeking to balance the promotion of practices considered beneficial with respect for the individual desires and choices of women.

This information is consistent with the findings in Vedam’s study on the integration of midwives in the United States, highlighting that qualified midwives can help a woman evaluate options for the method and place of childbirth, according to her health status, and thus facilitate access to appropriate resources. Ideally, they would practice in a legal setting, collaborating with other healthcare professionals in all childbirth settings [38].

Therefore, the clear limitation of studies available in the literature focusing on autonomy highlights the urgent need to develop research that specifically evaluates this aspect of assistance. Increasingly, autonomy of choice has been required in different areas of medicine, and in obstetrics the same can be observed more markedly. In the context of childbirth care, the lack of autonomy can be considered as a form of obstetric violence, which is another rising issue.

The experience of motherhood is characterized as a moment of great transformation and impact. In this sense, it is essential that women have their decisions respected, as their role is essential for them to live this experience in an active and participatory way. Being the protagonist in childbirth implies, among other aspects, having the appropriate and necessary knowledge to make decisions and choices.

Therefore, to remedy these gaps in the literature and improve the lack of knowledge necessary for women to have autonomy in making decisions about the method of childbirth, it is crucial to implement comprehensive educational strategies. This may include more comprehensive prenatal information programs, individualized counseling sessions, and the creation of accessible educational resources. Healthcare professionals, including obstetricians and midwives, play a key role in providing clear and understandable information, clarifying available options and promoting open communication. Additionally, encouraging women to participate in discussions about their childbirth plan and offering ongoing support throughout the process helps to enable them to make informed decisions and align their choices with their individual needs and desires.

## Conclusions

The literature has a small number of scientific articles that address the issue of respect by the care team for the autonomy of women in choosing the way of birth of their children at the time of giving birth.

In the review carried out, the available articles show that the inclusion of women in the process of choosing the method of childbirth is possible and that such choices did not alter the safety of birth. The authors showed that the ability to decide on care related to maternity and the choice of method of childbirth, although it is a woman’s right, is impacted by several elements. This includes the maternity care model, interactions with health professionals (the team that provides childbirth care), the influences of family and friends, in addition to the favorable or unfavorable experiences of the women themselves.

Therefore, the influence of the medical team or the obstetrician has a strong impact on decision-making power, because when women choose alone, most of them opt for vaginal childbirth. However, when faced with the recommendations of their doctors and care team, they accept the proposed indication, even though they do not know for sure why such an indication was made, deducing that this was a choice made to increase childbirth safety.

It was also clearly observed that this participation in choosing the method of childbirth has the capacity to increase women’s satisfaction with the birth process of their children, and this satisfaction with the childbirth and assistance received is validated by the literature through the application of the MADM and MOR scales, which assess the satisfaction and autonomy of women in maternity care.

The findings of this review present the role of midwives in choosing the method of childbirth and reveal an intricate ethical web. In this study, midwives prefer vaginal childbirth in keeping with the ethical principles of beneficence, avoiding unnecessary interventions. However, the midwives’ commitment to respecting women’s freedom of choice also stands out, highlighting the ethical principle of respect for autonomy. The duality between the "natural" perspective and respect for autonomy highlights the ethical complexity faced by midwives, who seek to balance the promotion of beneficial practices with respect for the individual wishes of women.

Therefore, it was concluded that pregnant women have the fundamental right to choose their method of childbirth. It is essential, however, that all women are properly guided throughout prenatal care, either by the obstetrician or the midwife, about the options, risks and benefits of each type of childbirth, respecting the ethical principle of beneficence, non-maleficence, justice and autonomy.

Future papers should be organized to evaluate the approach to women’s desires during prenatal care, in addition to also evaluating respect for the pregnant woman’s autonomy when she enters the maternity ward. New research must be carried out on the strategies of health professionals, obstetricians and midwives, to maintain effective communication and clarify the options available regarding the method of childbirth, helping women in their choice, making clear their role in building autonomy.

## Supporting information

S1 File Exemplary search string Pubmed. (DOCX)

S2 File Spreadsheets of the selection of articles. (DOCX)

S1 Table Data extraction instrument according to author and year, name of the article, location of the study, method, objective and conclusion. (DOCX)

S2 Table Articles selected for the study and the data extracted: Author and year, name of the article, location of the study, method, objective and conclusion. (DOCX)

S3 Table Preferred Reporting Items for Systematic reviews and Meta-Analyses extension for Scoping Reviews (PRISMA-ScR) checklist. (DOCX)
