# Supplementary material for: Pregnant women autonomy when choosing their method of childbirth: Scoping review
Source: PLoS One. 2024 Jul 11;19(7):e0304955. doi: 10.1371/journal.pone.0304955 (PMC11238978; doi:10.1371/journal.pone.0304955)
Supplement: S1 File — (DOCX) [file pone.0304955.s001.docx]

**File 1. Exemplary Search String Pubmed**

Search date: December 01, 2022 until November 30, 2023.

Search String Pubmed

(((((((((((((pregnant women[Title]) AND (delivery[Title])) AND (autonomy[Title])) OR (Parturients[MeSH Terms])) OR (Childbirth Assistance[MeSH Terms])) OR (Natural Childbirth[MeSH Terms])) OR (Cesarean Section[MeSH Terms])) OR (Patient Preferences[MeSH Terms])) AND (pregnant women[Title/Abstract])) AND (Childbirth Assistance[Title/Abstract])) AND (Midwifery[Title/Abstract])) AND (midwife[Title/Abstract])) AND (Humanized Delivery[Title/Abstract])) OR (Humanized Delivery[MeSH Terms])
