# Supplementary material for: Pregnant women autonomy when choosing their method of childbirth: Scoping review
Source: PLoS One. 2024 Jul 11;19(7):e0304955. doi: 10.1371/journal.pone.0304955 (PMC11238978; doi:10.1371/journal.pone.0304955)
Supplement: S2 File — (DOCX) [file pone.0304955.s002.docx]

**File 2. Spreadsheets of the selection of articles in the databases**

**Available at: 10.6084/m9.figshare.25529230**

**LILACS**

**keywords:** pregnant women; delivery; autonomy, childbirth assistance, parturients, midwifery, natural, childbirth, cesarean section, patient preferences, humanized delivery.

**Selection:** 06 articles 🡪 01 approved.

| **Article No.** | **Full Title of the Article** | **Authors** | **Journal** | **Year of Publication** | **Access Link** | **Status** |
| --- | --- | --- | --- | --- | --- | --- |
| 1 | Percepção de puérperas sobre a posição vertical no parto | Joelma Lacerda de Sousa;  Iolanda Pereira da Silva;  Lucimar Ramos Ribeiro Gonçalves;  Inez Sampaio Nery;  Ivanilda Sepúlveda Gomes;  Larissa Ferreira Cavalcante Sousa | Revista Baiana de Enfermagem | 2018 | [Link](http://www.revenf.bvs.br/scielo.php?script=sci_arttext&pid=S2178-86502018000100353) | REJECTED, OFF TOPIC |
| 2 | Aplicação das melhores práticas a mulheres grávidas no centro obstétrico | Bianca da Costa Vieira;  Marli Terezinha Stein Backes;  Lediana Dalla Costa;  Vanessa Martinhago Borges Fernandes;  Heloísa Helena Zimmer Ribas Dias;  Dirce Stein Backes | Revista Brasileira de Enfermagem | 2019 | [Link](http://www.revenf.bvs.br/scielo.php?script=sci_arttext&pid=S0034-71672019000900191) | REJECTED, OFF TOPIC |
| 3 | Autonomia da gestante na escolha do tipo de parto | Mônica Maria de Jesus Silva;  Semara Caroline Brandão Silva;  Gabriel Arruda Melo | Investigación en Enfermería: Imagen y Desarrollo | 2019 | [Link](https://revistas.javeriana.edu.co/index.php/imagenydesarrollo/article/view/19754) | APPROVED |
| 4 | Escolha informada no parto: um pensar para o cuidado centrado nas necessidades da mulher | Thelma Malagutti Sodré;  Miriam Aparecida Barbosa Merighi;  Isabel Cristina Bonadio | Ciência, cuidado e saúde | 2012 | [Link](https://periodicos.uem.br/ojs/index.php/CiencCuidSaude/article/view/17062/pdf) | REJECTED, NOT IN THE DELIMITED PERIOD |
| 5 | Escolha informada no parto: um pensar para o cuidado centrado nas necessidades da mulher | Thelma Malagutti Sodré;  Miriam Aparecida Barbosa Merighi;  Isabel Cristina Bonadio | Ciência, cuidado e saúde | 2012 | [Link](http://revista.cofen.gov.br/index.php/enfermagem/article/view/1333/480) | REJECTED, OFF TOPIC |
| 6 | Percepción social de usuarias atendidas exclusivamente por enfermeras en la etapa perinatal | Danelia Gómez Torres;  Gabriela Téllez Rojas; Pedro Miguel Santos Dinis Pareira;  Aida Maris Peres | Revista Enfermería Actual | 2018 | [Link](https://docs.bvsalud.org/biblioref/2019/04/953205/enf35n116.pdf) | REJECTED, OFF TOPIC |

**SCOPUS**

**keywords:** pregnant women; delivery; autonomy, childbirth assistance, parturients, midwifery, natural, childbirth, cesarean section, patient preferences, humanized delivery.

**Selection:** 61 articles 🡪 41 approved.

| **Article No.** | **Full Title of the Article** | **Authors** | **Journal** | **Year of Publication** | **Access Link** | **Status** |
| --- | --- | --- | --- | --- | --- | --- |
| 1 | Determinants of completing recommended antenatal care utilization in sub-Saharan from 2006 to 2018: evidence from 36 countries using Demographic and Health Surveys | Tessema, Z.T., Teshale, A.B., Tesema, G.A., Tamirat, K.S. | BMC Pregnancy and Childbirth | 2021 | Restricted access | REJECTED, OFF TOPIC |
| 2 | A qualitative study to explore the barriers for nonadherence to referral to hospital births by women with high-risk pregnancies in Nepal | Rajbanshi, S., Norhayati, M.N., Hazlina, N.H.N. | International Journal of Environmental Research and Public Health | 2021 | Restricted access | REJECTED, OFF TOPIC |
| 3 | Balancing risks: making decisions for maternal treatment without data on fetal safety | Minkoff, H., Ecker, J. | American Journal of Obstetrics and Gynecology | 2021 | Restricted access | REJECTED, OFF TOPIC |
| 4 | Maternal Capabilities Are Associated with Child Caregiving Behaviors Among Women in Rural Zimbabwe | Matare, C.R., Mbuya, M.N.N., Dickin, K.L., Constas, M.A., Pelto, G., Chasekwa, B., Humphrey, J.H., Stoltzfus, R.J., Sanitation Hygiene Infant Nutrition Efficacy (SHINE) Trial Team | The Journal of nutrition | 2021 | Restricted access | REJECTED, OFF TOPIC |
| 5 | Guideline No. 410: Prevention, Screening, Diagnosis, and Pregnancy Management for Fetal Neural Tube Defects | Douglas Wilson, R., Van Mieghem, T., Langlois, S., Church, P. | Journal of Obstetrics and Gynaecology Canada | 2021 | Restricted access | REJECTED, OFF TOPIC |
| 6 | Dimensions of women’s empowerment on access to skilled delivery services in Nepal | Khatiwada, J., Muzembo, B.A., Wada, K., Ikeda, S. | BMC Pregnancy and Childbirth | 2020 | Restricted access | REJECTED, OFF TOPIC |
| 7 | Legal and policy responses to the delivery of abortion care during COVID-19 | Romanis, E.C., Parsons, J.A. | International Journal of Gynecology and Obstetrics | 2020 | Restricted access | REJECTED, OFF TOPIC |
| 8 | Who safeguards pregnant women's autonomy during the COVID-19 pandemic? | Linden, K., Maimburg, R.D. | Sexual and Reproductive Healthcare | 2020 | Restricted access | REJECTED, OFF TOPIC |
| 9 | Use of Shared Decision-Making in Response to Maternal Request for Elective Cesarean Birth | Scaffidi, R.M., Padden-Denmead, M.L. | Journal of Midwifery and Women's Health | 2020 | Restricted access | REJECTED, OFF TOPIC |
| 10 | What are the implications of zika virus for infant feeding? A synthesis of qualitative evidence concerning congenital zika syndrome (czs) and comparable conditions | Carroll, C., Booth, A., Campbell, F., Relton, C. | PLoS Neglected Tropical Diseases | 2020 | Restricted access | REJECTED, OFF TOPIC |

| 11 | Measuring respect and autonomy in Dutch maternity care: Applicability of two measures | Feijen-de Jong, E.I., van der Pijl, M., Vedam, S., Jansen, D.E.M.C., Peters, L.L. | Women and Birth | 2020 | Restricted access | REJECTED, OFF TOPIC |
| --- | --- | --- | --- | --- | --- | --- |
| 12 | Response of an Obstetric Unit during the Coronavirus Disease of 2019 (COVID-19) Pandemic: Experiences from a Tertiary Care Center | Kumaraswami, S., Pradhan, T.S., Vrabie-Wolf, S., Lodhi, S., Rajendran, G.P., Tedjarati, S.S., Grimes, C.L. | AJP Reports | 2020 | Restricted access | REJECTED, OFF TOPIC |
| 13 | French ambulatory cesarean: Mother and newborn safety | Dimassi, K., Ami, O., Fauck, D., Simon, B., Velemir, L., Triki, A. | International Journal of Gynecology and Obstetrics | 2020 | Restricted access | REJECTED, OFF TOPIC |
| 14 | Anticipating issues with capacitous pregnant women: United Lincolnshire NHS Hospitals Trust v CD [2019] EWCOP 24 and Guys and St Thomas’ NHS Foundation Trust (GSTT) and South London and Maudsley NHS Foundation Trust (SLAM) v R [2020] EWCOP 4 | Fovargue, S. | Medical Law Review | 2020 | Restricted access | REJECTED, OFF TOPIC |
| 15 | Women's autonomy – A cornerstone of reproductive rights | Maimburg, R.D. | Sexual and Reproductive Healthcare | 2019 | Restricted access | REJECTED, OFF TOPIC |
| 16 | HOspital care versus TELemonitoring in high-risk pregnancy (HOTEL): Study protocol for a multicentre non-inferiority randomised controlled trial | Van Den Heuvel, J.F.M., Ganzevoort, W., De Haan-Jebbink, J.M., Van Der Ham, D.P., Deurloo, K.L., Seeber, L., Franx, A., Bekker, M.N. | BMJ Open | 2019 | Restricted access | REJECTED, OFF TOPIC |
| 17 | Planned home birth in Slovenia—Are we ready? | Takač, I., Belak, U., Gorjup, D., Kavšek, G., Macun, E., Medved, R., Mihevc Ponikvar, B., Mole, H., Mujezinović, F., Najdenov, P., Prelec, A., Premru Sršen, T., Mikluš, M., Serdinšek, T., Sobočan, M., Steblovnik, L., Tičar, Z., Horvat, M., Jamšek, T., Arko, D. | International Journal of Health Planning and Management | 2019 | Restricted access | REJECTED, OFF TOPIC |
| 18 | Respectful maternity care: A national landscape review | Ansari, H., Yeravdekar, R. | National Medical Journal of India | 2019 | Restricted access | REJECTED, OFF TOPIC |
| 19 | Narratives on why pregnant women delay seeking maternal health care during delivery and obstetric complications in rural Ghana | Sumankuuro, J., Mahama, M.Y., Crockett, J., Wang, S., Young, J. | BMC Pregnancy and Childbirth | 2019 | Restricted access | REJECTED, OFF TOPIC |
| 20 | Consent in pregnancy: A qualitative study of the views and experiences of women and their healthcare professionals | Nicholls, J., David, A.L., Iskaros, J., Lanceley, A. | European Journal of Obstetrics and Gynecology and Reproductive Biology | 2019 | Restricted access | REJECTED, OFF TOPIC |

| 21 | Introduction of new vaccines for immunization in pregnancy – Programmatic, regulatory, safety and ethical considerations | Kochhar, S., Edwards, K.M., Ropero Alvarez, A.M., Moro, P.L., Ortiz, J.R. | Vaccine | 2019 | Restricted access | APPROVED |
| --- | --- | --- | --- | --- | --- | --- |
| 22 | Differences in abortion attitudes by policy context and between men and women in the World Values Survey | Loll, D., Hall, K.S. | Women and Health | 2019 | Restricted access | APPROVED |
| 23 | Responding to evolving abortion regulations — The critical role of primary care | Beaman, J., Schillinger, D. | New England Journal of Medicine | 2019 | Restricted access | APPROVED |
| 24 | Can women in labor give informed consent to epidural analgesia? | Wada, K., Charland, L.C., Bellingham, G. | Bioethics | 2019 | Restricted access | APPROVED |
| 25 | Derivation and validation of a model predicting the likelihood of vaginal birth following labour induction | Alavifard, S., Meier, K., Shulman, Y., Tomlinson, G., D'Souza, R. | BMC Pregnancy and Childbirth | 2019 | Restricted access | APPROVED |
| 26 | Pearls in clinical obstetrics: Challenges in anticoagulation in pregnancy | della Torre, M., Sutherland, M.B., Digiovanni, L.M. | Minerva Ginecologica | 2019 | Restricted access | APPROVED |
| 27 | Response to ‘We need to treat pregnant women as adults’ | Petersen, R.W. | Australian and New Zealand Journal of Obstetrics and Gynaecology | 2019 | Restricted access | APPROVED |
| 28 | Barriers and outcomes associated with unfulfilled requests for permanent contraception following vaginal delivery | Flink-Bochacki, R., Flaum, S., Betstadt, S.J. | Contraception | 2019 | Restricted access | APPROVED |
| 29 | A Current Commentary on Breech Vaginal Deliveries: The Changing Landscape of Patient versus Physician Autonomy | Hill, M.G. | AJP Reports | 2019 | Restricted access | APPROVED |
| 30 | We need to treat pregnant women as adults: Women should be consented for an attempt at normal vaginal birth as for operative delivery, with risks and potential complications explained | Dietz, H.P., Callaghan, S. | Australian and New Zealand Journal of Obstetrics and Gynaecology | 2018 | Restricted access | APPROVED |

| 31 | How mandatory is the mandatory reporting of children at risk? | Isaacs, D., Britton, P.N., Kilham, H.A., Bag, S., Marks, S. | Journal of Paediatrics and Child Health | 2018 | Restricted access | APPROVED |
| --- | --- | --- | --- | --- | --- | --- |
| 32 | The challenges of institutionalizing community-level social accountability mechanisms for health and nutrition: A qualitative study in Odisha, India | Feruglio, F., Nisbett, N. | BMC Health Services Research | 2018 | Restricted access | APPROVED |
| 33 | Limits to the scope of non-invasive prenatal testing (NIPT): An analysis of the international ethical framework for prenatal screening and an interview study with Dutch professionals | Kater-Kuipers, A., Bunnik, E.M., De Beaufort, I.D., Galjaard, R.J.H. | BMC Pregnancy and Childbirth | 2018 | Restricted access | APPROVED |
| 34 | Pregnant women's recollections of early maternal bonding: associations with maternal–fetal attachment and birth choices | Handelzalts, J.E., Preis, H., Rosenbaum, M., Gozlan, M., Benyamini, Y. | Infant Mental Health Journal | 2018 | Restricted access | APPROVED |
| 35 | Women’s perspectives of mistreatment during childbirth at health facilities in Ghana: findings from a qualitative study | Maya, E.T., Adu-Bonsaffoh, K., Dako-Gyeke, P., Badzi, C., Vogel, J.P., Bohren, M.A., Adanu, R. | Reproductive Health Matters | 2018 | Restricted access | APPROVED |
| 36 | Ethical arguments for access to abortion services in the Republic of Ireland: Recent developments in the public discourse | McCarthy, J., O'Donnell, K., Campbell, L., Dooley, D. | Journal of Medical Ethics | 2018 | Restricted access | APPROVED |
| 37 | A revised version of the multidimensional health locus of control scales for labour and delivery (MHLC-LD-R) | Thege, B.K., Sallay, V., Rafael, B., Martos, T. | European Journal of Mental Health | 2018 | Restricted access | APPROVED |
| 38 | Choosing to Decline: Finding Common Ground through the Perspective of Shared Decision Making | Megregian, M., Nieuwenhuijze, M. | Journal of Midwifery and Women's Health | 2018 | Restricted access | APPROVED |
| 39 | Predictors of women's utilization of primary health care for skilled pregnancy care in rural Nigeria | Okonofua, F., Ntoimo, L., Ogungbangbe, J., Anjorin, S., Imongan, W., Yaya, S. | BMC Pregnancy and Childbirth | 2018 | Restricted access | APPROVED |
| 40 | Autonomy and infant feeding decision-making among teenage mothers in a rural and urban setting in KwaZulu-Natal, South Africa | Jama, N.A., Wilford, A., Haskins, L., Coutsoudis, A., Spies, L., Horwood, C. | BMC Pregnancy and Childbirth | 2018 | Restricted access | APPROVED |

| 41 | Experiences of gestational diabetes and gestational diabetes care: A focus group and interview study | Parsons, J., Sparrow, K., Ismail, K., Hunt, K., Rogers, H., Forbes, A. | BMC Pregnancy and Childbirth | 2018 | Restricted access | APPROVED |
| --- | --- | --- | --- | --- | --- | --- |
| 42 | Women's rights in the health care system: Caesarean delivery on maternal request | Larsen, A.-C. | Journal of Law and Medicine | 2018 | Restricted access | APPROVED |
| 43 | Rapid qualitative review of ethical issues surrounding healthcare for pregnant women or women of reproductive age in epidemic outbreaks | Hummel, P., Saxena, A., Klingler, C. | Epidemiology and health | 2018 | Restricted access | APPROVED |
| 44 | Midwives in England and in Italy: Two realities compared | Dell'Omodarme, C., Bocci, G., Troiano, G., Rossi, S., Kundisova, L., Nante, N., Alaimo, L. | Annali di Igiene | 2018 | Restricted access | APPROVED |
| 45 | A pilot exploratory investigation on pregnant women's views regarding STan fetal monitoring technology | Bryson, K., Wilkinson, C., Kuah, S., Matthews, G., Turnbull, D. | BMC Pregnancy and Childbirth | 2017 | Restricted access | APPROVED |
| 46 | Protected to death: Systematic exclusion of pregnant women from Ebola virus disease trials | Gomes, M.F., De La Fuente-Núñez, V., Saxena, A., Kuesel, A.C. | Reproductive Health | 2017 | Restricted access | APPROVED |
| 47 | The role of male partners in women's participation in research during pregnancy: A case study from the partners demonstration project | Ngure, K., Trinidad, S.B., Beima-Sofie, K., Baeten, J.M., Mugo, N.R., Bukusi, E.A., Heffron, R., John-Stewart, G., Kelley, M.C. | Reproductive Health | 2017 | Restricted access | APPROVED |
| 48 | Conceptualizing pathways linking women's empowerment and prematurity in developing countries | Afulani, P.A., Altman, M., Musana, J., Sudhinaraset, M. | BMC Pregnancy and Childbirth | 2017 | Restricted access | APPROVED |
| 49 | Mobile text messages-a silent revolution for a sound antenatal care | Indira, E., Thomas, P.E., Sri Hari, M. | Indian Journal of Public Health Research and Development | 2017 | Restricted access | APPROVED |
| 50 | Variation in clinical decision-making for induction of labour: A qualitative study | Nippita, T.A., Porter, M., Seeho, S.K., Morris, J.M., Roberts, C.L. | BMC Pregnancy and Childbirth | 2017 | Restricted access | APPROVED |

| 51 | Women's decision-making autonomy and utilisation of maternal healthcare services: Results from the Bangladesh Demographic and Health Survey | Ghose, B., Feng, D., Tang, S., Yaya, S., He, Z., Udenigwe, O., Ghosh, S., Feng, Z. | BMJ Open | 2017 | Restricted access | APPROVED |
| --- | --- | --- | --- | --- | --- | --- |
| 52 | Planned home birth | Walker, J.J. | Best Practice and Research: Clinical Obstetrics and Gynaecology | 2017 | Restricted access | APPROVED |
| 53 | The importance of clinically and ethically fine-tuning decision-making about cesarean delivery | Nguyen, M.T., McCullough, L.B., Chervenak, F.A. | Journal of Perinatal Medicine | 2017 | Restricted access | APPROVED |
| 54 | Montgomery and implications for clinical practice | Smith, A. | BJOG: An International Journal of Obstetrics and Gynaecology | 2017 | Restricted access | APPROVED |
| 55 | Through the client's eyes: Using narratives to explore experiences of care transfers during pregnancy, childbirth, and the neonatal period | van Stenus, C.M.V., Gotink, M., Boere-Boonekamp, M.M., Sools, A., Need, A. | BMC Pregnancy and Childbirth | 2017 | Restricted access | APPROVED |
| 56 | Eat Well Keep Active: Qualitative findings from a feasibility and acceptability study of a brief midwife led intervention to facilitate healthful dietary and physical activity behaviours in pregnant women | Warren, L., Rance, J., Hunter, B. | Midwifery | 2017 | Restricted access | APPROVED |
| 57 | Risk resistance: constructing home birth as morally responsible on an online discussion group | Fage-Butler, A.M. | Health, Risk and Society | 2017 | Restricted access | APPROVED |
| 58 | Immediate delivery or expectant management in gestational diabetes at term: the GINEXMAL randomised controlled trial | Alberico, S., Erenbourg, A., Hod, M., Yogev, Y., Hadar, E., Neri, F., Ronfani, L., Maso, G., Marcella, M., Steblovnik, L., Sketelj, A., Tomazic, M., Frusca, T., Zatti, S., Lojacono, A., Pagani, G., Maggino, T., Mello, G., Mecacci, F., Martini, E., Zanini, A., Andreotti, C., Tenore, A., Dallavalle, C. | BJOG: An International Journal of Obstetrics and Gynaecology | 2017 | Restricted access | APPROVED |
| 59 | Mode of delivery among Swedish midwives and obstetricians and their attitudes towards caesarean section | Sahlin, M., Andolf, E., Edman, G., Wiklund, I. | Sexual and Reproductive Healthcare | 2017 | Restricted access | APPROVED |
| 60 | Enhancing psychological wellbeing of women approaching the childbirth: A controlled study with a mobile application | Carissoli, C., Villani, D., Gasparri, D., Riva, G. | Annual Review of CyberTherapy and Telemedicine | 2017 | Restricted access | APPROVED |
| 61 | Why do women not adhere to advice on maternal referral in rural Tanzania? Narratives of women and their family members | Pembe, A.B., Mbekenga, C.K., Olsson, P., Darj, E. | Global Health Action | 2017 | Restricted access | APPROVED |

Parte superior do formulário

**SCIELO**

**keywords:** pregnant women; delivery; autonomy, childbirth assistance, parturients, midwifery, natural, childbirth, cesarean section, patient preferences, humanized delivery.

**Selection:** 15 articles 🡪 01 approved.

| **Article No.** | **Full Title of the Article** | **Authors** | **Journal** | **Year of Publication** | **Access Link** | **Status** |
| --- | --- | --- | --- | --- | --- | --- |
| 1 | Escolha da via de parto pela mulher: autonomia ou indução? | Fernandes, Juliana Azevedo; Campos, Gastão Wagner de Sousa; e Francisco, Priscila Maria Stolses Bergamo | SciELO - Saúde Pública | 2019 | [Link](https://scielosp.org/article/sdeb/2019.v43n121/406-416/) | APPROVED |
| 2 | Escolha da via de parto pela mulher: autonomia ou indução? | Fernandes, Juliana Azevedo; Campos, Gastão Wagner de Sousa; e Francisco, Priscila Maria Stolses Bergamo | Saúde debate | 2019 | [Link](https://www.scielo.br/scielo.php?script=sci_arttext&pid=S0103-11042019000200406&lang=pt) | REJECTED, REPEATED |
| 3 | Consulta preanalgésica del parto: un proyecto hecho realidad | V. Aceña Fabian, H. Vasquez Lobo, JL Ortega García e F. Neira Reina | Revista da Sociedade Espanhola de Dor | 2014 | [Link](https://scielo.isciii.es/scielo.php?script=sci_arttext&pid=S1134-80462014000600004&lang=pt) | REJECTED, OFF TOPIC |
| 4 | Escolha da via de parto: expectativa de gestantes e obstetras | Leguizamon Junior T, Steffani JA, Bonamigo EL | Revista Bioética | 2013 | [Link](https://www.scielo.br/scielo.php?script=sci_arttext&pid=S1983-80422013000300015&lang=pt) | REJECTED, NOT IN THE DELIMITED PERIOD |
| 5 | Aconselhamento em DST/Aids às gestantes que realizaram o teste anti-HIV na admissão para o parto:os sentidos de uma prática | Patrícia de Lima Fonseca  Jorge Alberto Bernstein Iriart | Interface - Comunic., Saude, Educ | 2012 | [Link](https://www.scielosp.org/article/icse/2012.v16n41/395-407/) | REJECTED, OFF TOPIC |
| 6 | Aconselhamento em DST/Aids às gestantes que realizaram o teste anti-HIV na admissão para o parto: os sentidos de uma prática | Patrícia de Lima Fonseca  Jorge Alberto Bernstein Iriart | Interface - Comunic., Saude, Educ | 2012 | [Link](https://www.scielo.br/scielo.php?script=sci_arttext&pid=S1414-32832012000200009&lang=pt) | REJECTED, REPEATED |
| 7 | Representações sociais e decisões das gestantes sobre a parturição: protagonismo das mulheres | Raquel da Rocha Pereira  Selma Cristina Franco  Nelma Baldin | Saúde Soc. São Paulo | 2011 | [Link](https://www.scielosp.org/article/sausoc/2011.v20n3/579-589/) | REJECTED, OFF TOPIC |
| 8 | Representações sociais e decisões das gestantes sobre a parturição: protagonismo das mulheres | Raquel da Rocha Pereira  Selma Cristina Franco  Nelma Baldin | Saúde Soc. São Paulo | 2011 | [Link](https://www.scielo.br/scielo.php?script=sci_arttext&pid=S0104-12902011000300005&lang=pt) | REJECTED, REPEATED |
| 9 | Perfil das gestantes de alto risco e a cogestão da decisão sobre a via de parto entre médico e gestante | Fernandes, Juliana Azevedo; Campos, Gastão Wagner de Sousa; e Francisco, Priscila Maria Stolses Bergamo | SciELO - Saúde Pública | 2019 | [Link](https://scielosp.org/article/sdeb/2019.v43n121/406-416/) | REJECTED, REPEATED |
| 10 | Percepção e ação das doulas no processo de humanização do parto | Grecia, Luana Marques Romano; Lopes Neto, David; Dias, Valéria Pacheco; Moisés, Mitsi Silva. | REME rev. min. enferm | 2019 | [Link](https://search.scielo.org/scielo.php?script=sci_arttext&pid=S1415-27622019000100253&lang=pt) | REJECTED, OFF TOPIC |
| 11 | Reflexividade, autonomia e consentimento. Uma análise das experiências de mulheres na busca por um parto fisiológico na Cidade de Buenos Aires | Mantilla, M. J., & Di Marco, M. H.. | Sex, Salud Soc | 2020 | [Link](https://www.scielo.br/scielo.php?script=sci_arttext&pid=S1984-64872020000200260) | REJECTED, OFF TOPIC |
| 12 | Complementary and integrative practices by doulas in maternities in Fortaleza (CE) and Campinas (SP), Brazil | Silva RM da, Jorge HMF, Matsue RY, Ferreira Junior AR, Barros NF | Saúde E Sociedade | 2016 | [Link](https://www.scielo.br/scielo.php?script=sci_arttext&pid=S0104-12902016000100108&lng=en&nrm=iso&tlng=en) | REJECTED, OFF TOPIC |
| 13 | Escolha da via de parto: expectativa de gestantes e obstetras | Leguizamon Junior T, Steffani JA, Bonamigo EL | Revista Bioética | 2013 | [Link](https://www.scielo.br/scielo.php?script=sci_arttext&pid=S1983-80422013000300015&lng=en&nrm=iso&tlng=pt) | REJECTED, NOT IN THE DELIMITED PERIOD |
| 14 | Aconselhamento em DST/AIDS às gestantes que realizaram o teste anti-HIV na admissão para o parto: os sentidos de uma prática | Fonseca, P. de L., & Iriart, J. A. | Interface (Botucatu) | 2012 | [Link](https://www.scielo.br/scielo.php?script=sci_arttext&pid=S1414-32832012000200009) | REJECTED, OFF TOPIC |
| 15 | Representações sociais e decisões das gestantes sobre a parturição: protagonismo das mulheres | Pereira R da R, Franco SC, Baldin N | Saúde e Sociedade | 2011 | [Link](https://www.scielo.br/scielo.php?script=sci_arttext&pid=S0104-12902011000300005) | REJECTED, NOT IN THE DELIMITED PERIOD |

**PUBMED**

**keywords:** pregnant women; delivery; autonomy, childbirth assistance, parturients, midwifery, natural, childbirth, cesarean section, patient preferences, humanized delivery.

**Selection:** 80 articles 🡪 11 approved.

| **Article No.** | **Full Title of the Article** | | **Authors** | | **Journal** | | **Year of Publication** | **Access Link** | | **Status** | | |
| --- | --- | --- | --- | --- | --- | --- | --- | --- | --- | --- | --- | --- |
| 1 | Women's Autonomy and Skilled Attendance During Pregnancy and Delivery in Nepal. | | Kc S, Neupane S. | | Matern Child Health J | | 2016 | [Link](https://pubmed.ncbi.nlm.nih.gov/26979612/) | | REJECTED, OFF TOPIC | | |
| 2 | Narratives on why pregnant women delay seeking maternal health care during delivery and obstetric complications in rural Ghana. | | Sumankuuro J, Mahama MY, Crockett J, Wang S, Young J. | | BMC Pregnancy Childbirth | | 2019 | [Link](https://pubmed.ncbi.nlm.nih.gov/31337348/) | | REJECTED, OFF TOPIC | | |
| 3 | Exploring First-time Pregnant Women's Motivations for Planning Vaginal Delivery: A Qualitative Study. | | Darsareh F, Aghamolaei T, Rajaei M, Madani A. | | Iran J Nurs Midwifery Res | | 2018 | [Link](https://pubmed.ncbi.nlm.nih.gov/30386397/) | | REJECTED, OFF TOPIC | | |
| 4 | We need to treat pregnant women as adults: Women should be consented for an attempt at normal vaginal birth as for operative delivery, with risks and potential complications explained. | | Dietz HP, Callaghan S. | | Aust N Z J Obstet Gynaecol | | 2018 | [Link](https://pubmed.ncbi.nlm.nih.gov/30536511/) | | REJECTED, OFF TOPIC | | |
| 5 | Perceptions and experiences of pregnant women about routine HIV testing and counselling in Ghimbi town, Ethiopia: a qualitative study. | | Mitiku I, Addissie A, Molla M. | | BMC Res Notes | | 2017 | [Link](https://pubmed.ncbi.nlm.nih.gov/28209187/) | | REJECTED, OFF TOPIC | | |
| 6 | Pregnant women's recollections of early maternal bonding: associations with maternal-fetal attachment and birth choices. | | Handelzalts JE, Preis H, Rosenbaum M, Gozlan M, Benyamini Y. | | Infant Ment Health J | | 2018 | [Link](https://onlinelibrary.wiley.com/doi/abs/10.1002/imhj.21731) | | REJECTED, OFF TOPIC | | |
| 7 | Women's decision-making autonomy and utilisation of maternal healthcare services: results from the Bangladesh Demographic and Health Survey. | | Ghose B, Feng D, Tang S, Yaya S, He Z, Udenigwe O, Ghosh S, Feng Z. | | BMJ Open | | 2017 | [Link](https://pubmed.ncbi.nlm.nih.gov/28882921/) | | REJECTED, OFF TOPIC | | |
| 8 | Experiences of gestational diabetes and gestational diabetes care: a focus group and interview study. | | Parsons J, Sparrow K, Ismail K, Hunt K, Rogers H, Forbes A. | | BMC Pregnancy Childbirth | | 2018 | [Link](https://pubmed.ncbi.nlm.nih.gov/29325518/) | | REJECTED, OFF TOPIC | | |
| 9 | Hospital care versus TELemonitoring in high-risk pregnancy (HOTEL): study protocol for a multicentre non-inferiority randomised controlled trial. | | van den Heuvel JFM, Ganzevoort W, De Haan-Jebbink JM, van der Ham DP, Deurloo KL, Seeber L, Franx A, Bekker MN. | | BMJ Open | | 2019 | [Link](https://pubmed.ncbi.nlm.nih.gov/31662396/) | | REJECTED, OFF TOPIC | | |
| 10 | Measuring respect and autonomy in Dutch maternity care: Applicability of two measures. | | Feijen-de Jong EI, van der Pijl M, Vedam S, Jansen DEMC, Peters LL. | | Women Birth | | 2020 | [Link](https://pubmed.ncbi.nlm.nih.gov/31796343/) | | APPROVED | | |
| 11 | Eat Well Keep Active: Qualitative findings from a feasibility and acceptability study of a brief midwife led intervention to facilitate healthful dietary and physical activity behaviours in pregnant women. | | Warren L, Rance J, Hunter B. | | Midwifery | | 2017 | [Link](https://pubmed.ncbi.nlm.nih.gov/27964858/) | | REJECTED, OFF TOPIC | | |
| 12 | Guideline No. 410: Prevention, Screening, Diagnosis, and Pregnancy Management for Fetal Neural Tube Defects. | | Douglas Wilson R, Van Mieghem T, Langlois S, Church P. | | J Obstet Gynaecol Can | | 2021 | [Link](https://pubmed.ncbi.nlm.nih.gov/33212246/) | | REJECTED, OFF TOPIC | | |
| 13 | Pearls in clinical obstetrics: challenges in anticoagulation in pregnancy. | | Della Torre M, Sutherland MB, Digiovanni LM. | | Minerva Ginecol | | 2019 | [Link](https://pubmed.ncbi.nlm.nih.gov/30360601/) | | REJECTED, OFF TOPIC | | |
| 14 | Conceptualizing pathways linking women's empowerment and prematurity in developing countries. | | Afulani PA, Altman M, Musana J, Sudhinaraset M. | | BMC Pregnancy Childbirth | | 2017 | [Link](https://pubmed.ncbi.nlm.nih.gov/29143627/) | | REJECTED, OFF TOPIC | | |
| 15 | Women's autonomy - A cornerstone of reproductive rights. | | Maimburg RD. | | Sex Reprod Healthc | | 2019 | [Link](https://pubmed.ncbi.nlm.nih.gov/31679989/) | | REJECTED, OFF TOPIC | | |
| 16 | [Prevention and detection of obstetric violence: A need in the Spanish delivery rooms?]. | | Freire Barja N, Luces Lago AM, Mosquera Pan L, Tizón Bouza E. | | Rev Enferm. | | 2016 | [Link](https://pubmed.ncbi.nlm.nih.gov/29584391/) | | REJECTED, OFF TOPIC | | |
| 17 | Factors associated with institutional delivery service utilization in Ethiopia. | | Kebede A, Hassen K, Nigussie Teklehaymanot A. | | Int J Womens Health. | | 2016 | [Link](https://pubmed.ncbi.nlm.nih.gov/27672342/) | | REJECTED, OFF TOPIC | | |
| 18 | Planned home birth in Slovenia-Are we ready? | | Takač I, Belak U, Gorjup D, Kavšek G, Macun E, Medved R, Mihevc Ponikvar B, Mole H, Mujezinović F, Najdenov P, Prelec A, Premru Sršen T, Mikluš M, Serdinšek T, Sobočan M, Steblovnik L, Tičar Z, Horvat M, Jamšek T, Arko D. | | Int J Health Plann Manage. | | 2019 | [Link](https://pubmed.ncbi.nlm.nih.gov/31436355/) | | REJECTED, OFF TOPIC | | |
| 19 | Anticipating Issues with Capacitous Pregnant Women: United Lincolnshire NHS Hospitals Trust v CD [2019] EWCOP 24 and Guys and St Thomas' NHS Foundation Trust (GSTT) and South London and Maudsley NHS Foundation Trust (SLAM) v R [2020] EWCOP 4. | | Fovargue S. | | Med Law Rev. | | 2020 | [Link](https://pubmed.ncbi.nlm.nih.gov/32810266/) | | REJECTED, OFF TOPIC | | |
| 20 | Respectful maternity care: A national landscape review. | | Ansari H, Yeravdekar R. | | Natl Med J India. | | 2019 | [Link](https://pubmed.ncbi.nlm.nih.gov/32985445/) | | REJECTED, OFF TOPIC | | |
| 21 | Derivation and validation of a model predicting the likelihood of vaginal birth following labour induction. | | Alavifard S, Meier K, Shulman Y, Tomlinson G, D'Souza R. | | BMC Pregnancy Childbirth. | | 2019 | [Link](https://pubmed.ncbi.nlm.nih.gov/30991983/) | | REJECTED, OFF TOPIC | | |
| 22 | Autonomy and infant feeding decision-making among teenage mothers in a rural and urban setting in KwaZulu-Natal, South Africa. | | Jama NA, Wilford A, Haskins L, Coutsoudis A, Spies L, Horwood C. | | BMC Pregnancy Childbirth. | | 2018 | [Link](https://pubmed.ncbi.nlm.nih.gov/29454323/) | | REJECTED, OFF TOPIC | | |
| 23 | Predictors of women's utilization of primary health care for skilled pregnancy care in rural Nigeria. | | Okonofua F, Ntoimo L, Ogungbangbe J, Anjorin S, Imongan W, Yaya S. | | BMC Pregnancy Childbirth. | | 2018 | [Link](https://pubmed.ncbi.nlm.nih.gov/29669538/) | | REJECTED, OFF TOPIC | | |
| 24 | A pilot exploratory investigation on pregnant women's views regarding STan fetal monitoring technology. | | Bryson K, Wilkinson C, Kuah S, Matthews G, Turnbull D. | | BMC Pregnancy Childbirth. | | 2017 | [Link](https://pubmed.ncbi.nlm.nih.gov/29284453/) | | REJECTED, OFF TOPIC | | |
| 25 | Women's perspectives of mistreatment during childbirth at health facilities in Ghana: findings from a qualitative study. | | Maya ET, Adu-Bonsaffoh K, Dako-Gyeke P, Badzi C, Vogel JP, Bohren MA, Adanu R. | | Reprod Health Matters. | | 2018 | [Link](https://pubmed.ncbi.nlm.nih.gov/30152268/) | | REJECTED, OFF TOPIC | | |
| 26 | | Psychosocial predictors of antenatal stress in Pakistan: perspectives from a developing country. | | Waqas A, Zubair M, Zia S, Meraj H, Aedma KK, Majeed MH, Naveed S. | | BMC Res Notes. | 2020 | | [Link](https://pubmed.ncbi.nlm.nih.gov/32188496/) | | REJECTED, OFF TOPIC |  |
| 27 | | Women's Rights in the Health Care System: Caesarean Delivery on Maternal Request. | | Larsen AC. | | J Law Med. | 2018 | | [Link](https://pubmed.ncbi.nlm.nih.gov/29978647/) | | APPROVED |  |
| 28 | | Rapid qualitative review of ethical issues surrounding healthcare for pregnant women or women of reproductive age in epidemic outbreaks. | | Hummel P, Saxena A, Klingler C. | | Epidemiol Health. | 2018 | | [Link](https://pubmed.ncbi.nlm.nih.gov/29370682/) | | REJECTED, OFF TOPIC |  |
| 29 | | Women׳s motivations for having unassisted childbirth or high-risk homebirth: An exploration of the literature on 'birthing outside the system'. | | Holten L, de Miranda E. | | Midwifery. | 2016 | | [Link](https://pubmed.ncbi.nlm.nih.gov/27055760/) | | REJECTED, OFF TOPIC |  |
| 30 | | [Moving beyond the ethical tension of caesarean section on maternal request]. | | Schantz C, Lhotte M, Pantelias AC. | | Sante Publique. | 2020 | | [Link](https://pubmed.ncbi.nlm.nih.gov/33723955/) | | REJECTED, OFF TOPIC |  |
| 31 | | Can women in labor give informed consent to epidural analgesia? | | Wada K, Charland LC, Bellingham G. | | Bioethics. | 2019 | | [Link](https://pubmed.ncbi.nlm.nih.gov/30358905/) | | REJECTED, OFF TOPIC |  |
| 32 | | Obstetric violence: a new framework for identifying challenges to maternal healthcare in Argentina | | Carlos HerreraVacaflor | |  | 2016 | | [Link](https://pubmed.ncbi.nlm.nih.gov/27578340/) | | REJECTED, OFF TOPIC |  |
| 33 | | Moral implications of obstetric technologies for pregnancy and motherhood. | | Susanne Brauer | |  | 2015 | | [Link](https://pubmed.ncbi.nlm.nih.gov/25837233/) | | REJECTED, OFF TOPIC |  |
| 34 | | A Violent Birth: Reframing Coerced Procedures During Childbirth as Obstetric Violence. | | Borges MT. | | Duke Law J. | 2018 | | [Link](https://pubmed.ncbi.nlm.nih.gov/29469554/) | | REJECTED, OFF TOPIC |  |
| 35 | | The challenges of institutionalizing community-level social accountability mechanisms for health and nutrition: a qualitative study in Odisha, India. | | Feruglio F, Nisbett N. | | BMC Health Serv Res. | 2018 | | [Link](https://pubmed.ncbi.nlm.nih.gov/30340490/) | | REJECTED, OFF TOPIC |  |
| 36 | | Effects of Payment for Performance on accountability mechanisms: Evidence from Pwani, Tanzania. | | Mayumana I, Borghi J, Anselmi L, Mamdani M, Lange S. | | Soc Sci Med. | 2017 | | [Link](https://pubmed.ncbi.nlm.nih.gov/28257886/) | | REJECTED, OFF TOPIC |  |

| 37 | Interventive Care: Uncertainty, Distributed Agency, and Cesarean Section in a Zika Virus Epidemic. | Williamson KE. | Med Anthropol Q. | 2020 | [Link](https://pubmed.ncbi.nlm.nih.gov/33174644/) | REJECTED, OFF TOPIC |
| --- | --- | --- | --- | --- | --- | --- |
| 38 | Utilization of maternal health care services and their determinants in Karnataka State, India. | Vidler M, Ramadurg U, Charantimath U, Katageri G, Karadiguddi C, Sawchuck D, Qureshi R, Dharamsi S, Joshi A, von Dadelszen P, Derman R, Bellad M, Goudar S, Mallapur A; Community Level Interventions for Pre-eclampsia (CLIP) India Feasibility Working Group. | Reprod Health. | 2016 | [Link](https://pubmed.ncbi.nlm.nih.gov/27356502/) | REJECTED, OFF TOPIC |
| 39 | Balancing risks: making decisions for maternal treatment without data on fetal safety. | Minkoff H, Ecker J. | Am J Obstet Gynecol. | 2021 | [Link](https://pubmed.ncbi.nlm.nih.gov/33539824/) | REJECTED, OFF TOPIC |
| 40 | Responding to refusal of recommended cesarean section: Promoting good parenting. | Malek J. | Semin Perinatol. | 2016 | [Link](https://pubmed.ncbi.nlm.nih.gov/26803168/) | REJECTED, OFF TOPIC |
| 41 | Through the client's eyes: using narratives to explore experiences of care transfers during pregnancy, childbirth, and the neonatal period. | van Stenus CMV, Gotink M, Boere-Boonekamp MM, Sools A, Need A. | BMC Pregnancy Childbirth. | 2017 | [Link](https://pubmed.ncbi.nlm.nih.gov/28606067/) | REJECTED, OFF TOPIC |
| 42 | Maternal Capabilities Are Associated with Child Caregiving Behaviors Among Women in Rural Zimbabwe. | Matare CR, Mbuya MNN, Dickin KL, Constas MA, Pelto G, Chasekwa B, Humphrey JH, Stoltzfus RJ; Sanitation Hygiene Infant Nutrition Efficacy (SHINE) Trial Team. | J Nutr. | 2021 | [Link](https://pubmed.ncbi.nlm.nih.gov/33211881/) | REJECTED, OFF TOPIC |
| 43 | Maternal request caesareans and COVID-19: the virus does not diminish the importance of choice in childbirth. | Romanis EC, Nelson A. | J Med Ethics. | 2020 | [Link](https://pubmed.ncbi.nlm.nih.gov/32913116/) | REJECTED, OFF TOPIC |
| 44 | Why the Elective Caesarean Lottery is Ethically Impermissible. | Romanis EC. | Health Care Anal. | 2019 | [Link](https://pubmed.ncbi.nlm.nih.gov/31037420/) | APPROVED |
| 45 | The Ethics of Court-Mandated Cesarean Sections. | Glezer A. | J Am Acad Psychiatry Law. | 2018 | [Link](https://pubmed.ncbi.nlm.nih.gov/30368459/) | REJECTED, OFF TOPIC |
| 46 | Choosing When to Be Born. | Illuzzi JL. | J Womens Health (Larchmt). | 2018 | [Link](https://pubmed.ncbi.nlm.nih.gov/29768113/) | REJECTED, OFF TOPIC |
| 47 | Responding professionally to requests for cesarean delivery. | Chervenak F, McCullough L. | Georgian Med News. | 2017 | [Link](https://pubmed.ncbi.nlm.nih.gov/28820404/) | REJECTED, OFF TOPIC |

| 48 | Continuous support for women during childbirth. | Bohren MA, Hofmeyr GJ, Sakala C, Fukuzawa RK, Cuthbert A. | Cochrane Database Syst Rev. | 2017 | [Link](https://pubmed.ncbi.nlm.nih.gov/28681500/) | REJECTED, OFF TOPIC |
| --- | --- | --- | --- | --- | --- | --- |
| 49 | Women's autonomy in the process of labour and childbirth: integrative literature review. | Reis TLDRD, Padoin SMM, Toebe TRP, Paula CC, Quadros JS. | Rev Gaucha Enferm. | 2017 | [Link](https://pubmed.ncbi.nlm.nih.gov/28443976/) | REJECTED, OFF TOPIC |
| 50 | The importance of clinically and ethically fine-tuning decision-making about cesarean delivery. | Nguyen MT, McCullough LB, Chervenak FA. | J Perinat Med. | 2017 | [Link](https://pubmed.ncbi.nlm.nih.gov/27780155/) | REJECTED, OFF TOPIC |
| 51 | Responding to refusal of recommended cesarean section: Promoting good parenting. | Malek J. | Semin Perinatol. | 2016 | [Link](https://pubmed.ncbi.nlm.nih.gov/26803168/) | REJECTED, REPEATED |
| 52 | Married women's autonomy and post-delivery modern contraceptive use in the Democratic Republic of Congo. | Sano Y, et al. | BMC Womens Health. | 2018 | [Link](https://pubmed.ncbi.nlm.nih.gov/29530032/) | REJECTED, OFF TOPIC |
| 53 | Dimensions of women's empowerment on access to skilled delivery services in Nepal. | Khatiwada J, et al. | BMC Pregnancy Childbirth. | 2020 | [Link](https://pubmed.ncbi.nlm.nih.gov/33059624/) | REJECTED, OFF TOPIC |
| 54 | An investigation of the relationship between autonomy, childbirth practices, and obstetric fistula among women in rural Lilongwe District, Malawi. | Kaplan JA, et al. | BMC Int Health Hum Rights. | 2017 | [Link](https://pubmed.ncbi.nlm.nih.gov/28629455/) | REJECTED, OFF TOPIC |
| 55 | Utilization of Deworming Drugs and Its Individual and Community Level Predictors among Pregnant Married Women in Cameroon: A Multilevel Modeling. | Zegeye B, et al. | Biomed Res Int. | 2021 | [Link](https://pubmed.ncbi.nlm.nih.gov/34095307/) | REJECTED, OFF TOPIC |
| 56 | Is it the decision of women to choose a cesarean section as the mode of birth? A review of literature on the views of stakeholders. | Loke AY, et al. | BMC Pregnancy Childbirth. | 2019 | [Link](https://pubmed.ncbi.nlm.nih.gov/31399072/) | REJECTED, REVIEW ARTICLE |
| 57 | Outpatient labour induction. | Wilkinson C. | Best Pract Res Clin Obstet Gynaecol. | 2021 | [Link](https://pubmed.ncbi.nlm.nih.gov/34556409/) | REJECTED, OFF TOPIC |

| 58 | [Breastfeeding protection according to the law on the reform of maternity protection]. | Arndt M, et al. | | Bundesgesundheitsblatt Gesundheitsforschung Gesundheitsschut | 2018 | [Link](https://pubmed.ncbi.nlm.nih.gov/29934680/) | REJECTED, OFF TOPIC |
| --- | --- | --- | --- | --- | --- | --- | --- |
| 59 | Management of thyrotoxicosis: preconception, pregnancy, and the postpartum period. | Pearce EN. | | Endocr Pract. | 2019 | [Link](https://pubmed.ncbi.nlm.nih.gov/30289300/) | REJECTED, OFF TOPIC |
| 60 | French ambulatory cesarean: Mother and newborn safety. | Dimassi K, et al. | | Int J Gynaecol Obstet. | 2020 | [Link](https://pubmed.ncbi.nlm.nih.gov/31642513/) | REJECTED, OFF TOPIC |
| 61 | Male involvement in promotion of safe motherhood in low- and middle-income countries: A scoping review. | Ladur AN, et al. | | Midwifery. | 2021 | [Link](https://pubmed.ncbi.nlm.nih.gov/34293604/) | REJECTED, OFF TOPIC |
| 62 | Barriers and outcomes associated with unfulfilled requests for permanent contraception following vaginal delivery. | Flink-Bochacki R, et al. | | Contraception. | 2019 | [Link](https://pubmed.ncbi.nlm.nih.gov/30465753/) | REJECTED, OFF TOPIC |
| 63 | Whose decision? Caesarean section and women with physical disabilities in Northern Vietnam: A qualitative study. | Nguyen TV, et al. | | Midwifery. | 2022 | [Link](https://pubmed.ncbi.nlm.nih.gov/34740027/) | REJECTED, OFF TOPIC |
| 64 | Childbirth experience of women in a maternity hospital signatory of the Adequate Childbirth Project: mixed study. | Barbosa LC, et al. | | Rev Gaucha Enferm. | 2021 | [Link](https://pubmed.ncbi.nlm.nih.gov/34755799/) | REJECTED, OFF TOPIC |
| 65 | Consent on the labour ward: A qualitative study of the views and experiences of healthcare professionals. | Kennedy S, et al. | | Eur J Obstet Gynecol Reprod Biol. | 2021 | [Link](https://pubmed.ncbi.nlm.nih.gov/34303075/) | REJECTED, OFF TOPIC |
| 66 | Male participation in antenatal care and its influence on their pregnant partners' reproductive health care utilization: insight from the 2015 Afghanistan Demographic and Health Survey. | Alemi S, et al. | | J Biosoc Sci. | 2021 | [Link](https://pubmed.ncbi.nlm.nih.gov/32536350/) | REJECTED, OFF TOPIC |
| 67 | African American Women's Experiences with Birth After a Prior Cesarean Section. | Miller MW, et al. | | Matern Child Health J. | 2021 | [Link](https://pubmed.ncbi.nlm.nih.gov/34731358/) | APPROVED |
| 68 | Autonomia e tomada de decisão de alimentação infantil entre mães adolescentes em um ambiente rural e urbano em KwaZulu-Natal, África do Sul. | Jama NA, et al. | | BMC Gravidez Parto. | 2018 | [Link](https://pubmed.ncbi.nlm.nih.gov/29454323/) | REJECTED, OFF TOPIC |
| 69 | The extraperitoneal French AmbUlatory cesarean section technique leads to improved pain scores and a faster maternal autonomy compared with the intraperitoneal Misgav Ladach technique: A prospective randomized controlled trial. | Dimassi K, et al. | | PLoS One. | 2021 | [Link](https://pubmed.ncbi.nlm.nih.gov/33481875/) | REJECTED, OFF TOPIC |
| 70 | Continuity of maternal healthcare services utilisation in Indonesia: analysis of determinants from the Indonesia Demographic and Health Survey. | Andriani H, et al. | | Fam Med Community Health. | 2021 | [Link](https://pubmed.ncbi.nlm.nih.gov/34937797/) | REJECTED, OFF TOPIC |
| 71 | Giving birth: Expectations of first time mothers in Switzerland at the mid point of pregnancy. | Fleming V, et al. | Women Birth. | | 2017 | [Link](https://pubmed.ncbi.nlm.nih.gov/28576618/) | REJECTED, OFF TOPIC |
| 72 | Exploring experiences and expectations of prenatal health care and genetic counseling/testing in immigrant Latinas. | Garza G, et al. | J Genet Couns. | | 2020 | [Link](https://pubmed.ncbi.nlm.nih.gov/32302061/) | REJECTED, OFF TOPIC |
| 73 | A Challenging Cesarean Delivery and Perioperative Course in a Former Polysubstance Abuser Without the Use of Traditional Opioids. | Gutman DA, et al. | J Med Cases. | | 2020 | [Link](https://pubmed.ncbi.nlm.nih.gov/34434365/) | REJECTED, OFF TOPIC |

| 74 | A national survey of Australian midwives' birth choices and outcomes | S Coulton Stoliar,  H G Dahlen ,  A Sheehan | Women Birth | 2023 | [Link](https://pubmed.ncbi.nlm.nih.gov/35927213/) | APPROVED |
| --- | --- | --- | --- | --- | --- | --- |
| 75 | Caesarean Section on Maternal Request-Ethical and Juridic Issues: A Narrative Review | Sorrentino F, Greco F, Palieri T, Vasciaveo L, Stabile G, Carlucci S, Laganà AS, Nappi L. | Medicina | 2022 | [Link](https://www.ncbi.nlm.nih.gov/pmc/articles/PMC9506057/) | APPROVED |
| 76 | Why women chose unassisted home birth in Malaysia: a qualitative study | Ahmad Tajuddin NAN, Suhaimi J, Ramdzan SN, Malek KA, Ismail IA, Shamsuddin NH, Abu Bakar AI, Othman S. | BMC Pregnancy Childbirth | 2020 | [Link](https://pubmed.ncbi.nlm.nih.gov/32429857/) | APPROVED |
| 77 | Moving beyond the ethical tension of caesarean section on maternal request | Schantz C, Lhotte M, Pantelias AC. | Sante Publique | 2020 | [Link](https://pubmed.ncbi.nlm.nih.gov/33723955/) | APPROVED |
| 78 | Patient-led decision making: Measuring autonomy and respect in Canadian maternity care | Vedam S, Stoll K, McRae DN, Korchinski M, Velasquez R, Wang J, Partridge S, McRae L, Martin RE, Jolicoeur G; CCinBC Steering Committee. | Patient Educ Couns. | 2019 | [Link](https://pubmed.ncbi.nlm.nih.gov/30448044/) | APPROVED |
| 79 | The Mother’s Autonomy in Decision Making (MADM) scale: Patient-led development and psychometric testing of a new instrument to evaluate experience of maternity care | Vedam S, Stoll K, Martin K, Rubashkin N, Partridge S, Thordarson D, Jolicoeur G; Changing Childbirth in BC Steering Council. | PLoS One | 2017 | [Link](https://www.ncbi.nlm.nih.gov/pmc/articles/PMC5322919/) | APPROVED |
| 80 | The Mothers on Respect (MOR) index: measuring quality, safety, and human rights in childbirth | Vedam S, Stoll K, Martin K, Rubashkin N, Partridge S, Thordarson D, Jolicoeur G; Changing Childbirth in BC Steering Council. | PLoS One | 2017 | [Link](https://www.ncbi.nlm.nih.gov/pmc/articles/PMC5322919/) | APROVADO |

Parte superior do formulário

**WEB OF SCIENCE**

**keywords:** pregnant women; delivery; autonomy, childbirth assistance, parturients, midwifery, natural, childbirth, cesarean section, patient preferences, humanized delivery.

**Selection:** 17 articles 🡪 02 approved.

| **Article No.** | **Full Title of the Article** | **Authors** | **Journal** | **Year of Publication** | **Access Link** | **Status** |
| --- | --- | --- | --- | --- | --- | --- |
| 1 | The attitudes and beliefs of Australian midwives and obstetricians about birth options and labor intervention | Dominiek Coates PhD, Natasha Donnolley PhD, Amanda Henry PhD | Journal of Midwifery and Women’s health | 2020 | [Link](https://onlinelibrary.wiley.com/doi/10.1111/jmwh.13168) | REJECTED, OFF TOPIC |
| 2 | Revisiting the Care Pathway for Trial of Labour After Cesarean: The Decision-to-Delivery Interval Is Key | R. Douglas Wilson, MD, MSc  Jessica Dy, MD, MPH  Jon Barrett, MD  Radha Chari, MD  Jennifer Blake, MD  B. Anthony Armson, MD | Journal of Obstetrics and Gynaecology Canadá | 2020 | [Link](https://www.jogc.com/article/S1701-2163(20)30693-9/fulltext) | REJECTED, OFF TOPIC |
| 3 | Determining factors of institutional delivery in India: A study from National Family Health Survey-4 (2015-16) | Bikash Barman, Avijit Roy, Ankita Zaveri, Jay Saha, Pradip Chouhan | Clinical Epidemiology and Global Health | 2020 | [Link](https://www.sciencedirect.com/science/article/pii/S2213398420301391) | REJECTED, OFF TOPIC |
| 4 | A Qualitative Comparison of Long- and Short-acting Hormonal Method: Users' Perspectives on Method Selection in Rural Guatemala | Kirsten Austad, Pooja Shah, Hannah Shryer, Peter Rohloff, Anita Chary | International Journal of Women's Health and Reproduction Sciences | 2020 | [Link](http://dx.doi.org/10.15296/ijwhr.2020.55) | REJECTED, OFF TOPIC |
| 5 | Women's experience of agency and respect in maternity care by type of insurance in California | Eugene Declercq ,Carol Sakala,Candice Belanoff | Plos One | 2020 | [Link](https://journals.plos.org/plosone/article?id=10.1371/journal.pone.0) | REJECTED, OFF TOPIC |
| 6 | Listening to Women: Understanding and Challenging Systems of Power to Achieve Reproductive Justice in South Carolina | Ellie Smith, Beth Sundstrom, Cara Delay | Journal of Social Issues | 2020 | [Link](https://spssi.onlinelibrary.wiley.com/doi/abs/10.1111/josi.12378) | REJECTED, OFF TOPIC |
| 7 | Breech Presentation: CNGOF Guidelines for Clinical Practice - Short Text | L. Sentilhes, T. Schmitz, E. Azria, D. Gallot, G. Ducarme, D. Korb, A. Mattuizzi, O. Parant, N. Sananès, S. Baumann, P. Rozenberg, M.-V. Senat, É. Verspyck | Gynécologie Obstétrique Fertilité & Sénologie | 2020 | [Link](https://www.sciencedirect.com/science/article/pii/S2468718919303393?v) | REJECTED, OFF TOPIC |
| 8 | Why the Elective Caesarean Lottery is Ethically Impermissible | Elizabeth Chloe Romanis | Health Care Anal | 2019 | [Link](https://link.springer.com/article/10.1007/s10728-019-00370-0) | APPROVED |
| 9 | Is it the decision of women to choose a cesarean section as the mode of birth? A review of literature on the views of stakeholders | Alice Yuen Loke, Louise Davies & Yim-wah Mak | BMC Pregnancy and Childbirth | 2019 | [Link](https://bmcpregnancychildbirth.biomedcentral.com/articles/10.1186/s12884-019-2440-2) | REJECTED, SYSTEMATIC REVIEW ON THE TOPIC |
| 10 | Consent in pregnancy: A qualitative study of the views and experiences of women and their healthcare professionals | Jacqueline Nicholls  Anna L David  Joseph Iskaros  Anne Lanceley | European Journal of Obstetrics & Gynecology and Reproductive Biology | 2019 | [Link](https://www.ejog.org/article/S0301-2115(19)30228-3/fulltext) | REJECTED, OFF TOPIC |

| 11 | Pearls in clinical obstetrics: challenges in anticoagulation in pregnancy | Della torre, Micaela & Sutherland, Monique & Digiovanni, Laura | Minerva Ginecologica | 2019 | [Link](https://www.researchgate.net/publication/331877766_Pearls_in_clinical_obstetrics_Challenges_in_anticoagulation_in_pregnancy) | REJECTED, OFF TOPIC |
| --- | --- | --- | --- | --- | --- | --- |
| 12 | Management of thyrotoxicosis: preconception, pregnancy, and the postpartum period | Elizabeth N. Pearce, | Endocrine Practice | 2019 | [Link](https://linkinghub.elsevier.com/retrieve/pii/S1530891X20359413) | REJECTED, OFF TOPIC |
| 13 | Pregnant women's recollections of early maternal bonding: associations with maternal-fetal attachment and birth choices | Jonathan E. Handelzalts, Heidi Preis, Maya Rosenbaum, Miri Gozlan, Yael Benyamini | Infant Mental Health Journal | 2018 | [Link](https://onlinelibrary.wiley.com/doi/abs/10.1002/imhj.21731) | REJECTED, OFF TOPIC |
| 14 | 'I didn't think you were allowed that, they didn't mention that.'A qualitative study exploring women's perceptions of home birth | Jo Naylor Smith, Beck Taylor, Karen Shaw, Alistair Hewison & Sara Kenyon | BMC Pregnancy and Childbirth | 2018 | [Link](https://bmcpregnancychildbirth.biomedcentral.com/articles/10.1186/s12884-018-1733-1) | REJECTED, OFF TOPIC |
| 15 | Autonomy and infant feeding decision-making among teenage mothers in a rural and urban setting in KwaZulu-Natal, South Africa | Ngcwalisa Amanda Jama, Aurene Wilford, Lyn Haskins, Anna Coutsoudis, Lenore Spies & Christiane Horwood | BMC Pregnancy and Childbirth | 2018 | [Link](https://bmcpregnancychildbirth.biomedcentral.com/articles/10.1186/s12884-018-1675-7) | REJECTED, OFF TOPIC |
| 16 | Women's motivations for choosing a high risk birth setting against medical advice in the Netherlands: a qualitative analysis | Martine Hollander, Esteriek de Miranda, Jeroen van Dillen, Irene de Graaf, Frank Vandenbussche & Lianne Holten | BMC Pregnancy and Childbirth | 2017 | [Link](https://bmcpregnancychildbirth.biomedcentral.com/articles/10.1186/s12884-017-1621-0) | REJECTED, OFF TOPIC |
| 17 | The Mother's Autonomy in Decision Making (MADM) scale: Patient-led development and psychometric testing of a new instrument to evaluate experience of maternity care | Vedam S, Stoll K, Martin K, Rubashkin N, Partridge S, Thordarson D, Jolicoeur G; Changing Childbirth in BC Steering Council. | Plos One | 2017 | [Link](https://journals.plos.org/plosone/article?id=10.1371/journal.pon) | APPROVED |

Parte superior do formulário

**Selection of articles in databases**

|  | **Approved** | **Off topic** | **Repeated** | **Revision** | **Out of date** | **TOTAL** |
| --- | --- | --- | --- | --- | --- | --- |
| **Pubmed**  **(N 80)** | 11 | 67 | 1 | 1 | 0 | 80 |
| **Scielo**  **(N 15)** | 1 | 7 | 4 | 0 | 3 | 15 |
| **Lilacs**  **(N 06)** | 1 | 4 | 0 | 0 | 1 | 06 |
| **Scopus**  **(N 61)** | 41 | 20 | 0 | 0 | 0 | 61 |
| **Web of Science**  **(N 17)** | 2 | 14 | 0 | 1 | 0 | 17 |
| TOTAL | 56 | 112 | 5 | 2 | 4 | 179 |
