# Supplementary material for: Pregnant women autonomy when choosing their method of childbirth: Scoping review
Source: PLoS One. 2024 Jul 11;19(7):e0304955. doi: 10.1371/journal.pone.0304955 (PMC11238978; doi:10.1371/journal.pone.0304955)
Supplement: S1 Table — (DOCX) [file pone.0304955.s003.docx]

Table 2. Data extraction instrument according to author and year, name of the article, location of the study, method, objective and conclusion.

| **Author and year** | **Name of the article** | **Location of the study** | **Method** | **Objective** | **Conclusion** |
| --- | --- | --- | --- | --- | --- |
|  |  |  |  |  |  |
