# Supplementary material for: Pregnant women autonomy when choosing their method of childbirth: Scoping review
Source: PLoS One. 2024 Jul 11;19(7):e0304955. doi: 10.1371/journal.pone.0304955 (PMC11238978; doi:10.1371/journal.pone.0304955)
Supplement: S2 Table — (DOCX) [file pone.0304955.s004.docx]

**Table 3.**

**Articles selected for the study and the data extracted: author and year, name of the article, location of the study, method, objective and conclusion.**

| **Author and year** | **Name of the article** | **Location of the study** | **Method** | **Objective** | **Conclusion** |
| --- | --- | --- | --- | --- | --- |
| Nguyen  (2016) | The importance of clinically and ethically  fine-tuning decision-making about cesarean  delivery | New York, United States of America | Literature review with preparation of an ethical clinical document. | Develop a clinical ethical framework with clinical and organizational factors for decision-making and advice on cesarean birth. | The adoption of a clinical ethical framework in practice to increase the autonomy of pregnant women by providing counseling to improve their autonomy in choosing the method of birth. |
| Vedam  (2017) | The Mother’s Autonomy in Decision Making (MADM) scale: Patient-led development and psychometric testing of a new instrument to evaluate experience of maternity care | British Columbia, Canada | Cross-sectional quantitative study applying the MADM scale with 2051 women | Develop and validate a new instrument that assesses women's autonomy and role in decision-making during maternity care. | The Mothers' Autonomy in Decision Making (MADM) scale proves to be reliable and valid for the decision-making process in maternity care. |
| Vedam  (2017) | The Mothers on Respect (MOR) index: measuring quality, safety, and human rights in childbirth | British Columbia, Canada | Cross-sectional study applying the MOR index with 1,672 women | Develop a survey tool that assesses women's experiences with maternity care, including their autonomy. | The MOR index is a reliable quality and safety indicator that can be applied across jurisdictions to assess access to autonomy-respecting maternity care. |
| Reis  (2017) | Female autonomy in the labor and birth process birth process: an integrative literature review | Rio Grande do Sul, Brazil | Integrative literature review. With article selection criteria | Identify the evidence available in scientific production about health care practices that interfere with the exercise of autonomy by Brazilian women in the labor and birth process. | There is a setback in the complete recognition and realization of women's rights, making it impossible to exercise their autonomy in relation to their own bodies and childbirth. |
| Bohren (2017) | Continuous support for women during childbirth | Geneva, Switzerland | Systematic literature review. With article selection criteria | Evaluate the effects of continuous and individual support when giving birth | Continued support during labor can improve outcomes for women, including increased spontaneous vaginal birth, shorter duration of labor, and decreased cesarean birth. |
| Vedam  (2019) | Patient-led decision making: Measuring autonomy and respect in Canadian maternity care | British Columbia, Canada | Cross-sectional online study applying the MADM and MOR scales exploring new topics with 2051 women. | Explore women's preferences and role in decision-making related to maternity care | Women's autonomy is significantly altered by the maternity care model, the nature of interactions with caregivers, and women's capacity for self-determination. |
| Fernandes  (2019) | Profile of high-risk pregnant women and co-management of the decision on the routh of birth delivery between doctor and pregnant woman | São Paulo,  Brazil | Cross-sectional, prospective study.  With a questionnaire applied to 405 pregnant women. | Characterize the profile of high-risk pregnant women when deciding on the method of birth. | When women choose their birth individually, the majority choose a natural birth. When only the doctor decides, the doctor recommends a cesarean section. And when it is decided together, a cesarean section prevails. |
| Loke  (2019) | Is it the decision of women to choose a cesarean section as the mode of birth? A review of literature on the views of stakeholders | Hong Kong, China | Systematic literature review. With article selection criteria | To explore the decision of women with low-risk pregnancies to undergo a cesarean section as a method of birth. | The decision-making process about the method of delivery is not simple and although the woman has autonomy, this can be influenced by the obstetrician, family and friends. |
| Feijen-de Jong (2020) | Measuring respect and autonomy in Dutch maternity care: Applicability of two measures | Groningen,  The Netherlands | Cross-sectional study applying the MADM and MOR scales with 557 pregnant women | Assess the applicability of Canadian measures; the Mothers' Autonomy in Decision Making (MADM) scale and the Mothers on Respect (MOR) index measures among pregnant women in the Netherlands | They support the feasibility, reliability and validity of applying the MOR and MADM scales to assess women's autonomy. |
| Tajuddin  (2020) | Why women chose unassisted home birth in Malaysia: a qualitative study | Kuala Lumpur, Malaysia | Prospective observational study using a questionnaire with 12 pregnant women | Exploring women's autonomy in relation to childbirth in Malaysia. | Women in Malaysia have complete autonomy to choose a natural home birth, being able to express their opinions and personal values to the detriment of health risks. |
| Schantz  (2020) | Moving beyond the ethical tension of caesarean section on maternal request | Paris, France | Prospective observational study using a questionnaire with 37 women (pregnant women and midwives) | Assess whether it is ethically acceptable for midwives to accompany a woman in her decision to have a cesarean section | Most women and midwives share a view of childbirth as something “natural”, preferring vaginal birth. This choice incorporates the ethical principles of beneficence and non-maleficence. On the other hand, midwives express the desire to respect the pregnant woman's choice and freedom, illustrating the ethical principle of respect for autonomy. |
| Miller  (2022) | African American Women's Experiences with Birth After a Prior Cesarean Section | North Carolina, United States of America | Prospective observational study using a questionnaire with 25 pregnant women | Characterize the pregnancy experience and decision about method of birth in African-American women with a previous cesarean section | It encourages shared decision-making between pregnant women and the medical team. |
| Zewude (2022) | The Preferences of Modes of Child Delivery and Associated Factors Among Pregnant Women in Southern Ethiopia | Sodo, Ethiopia | Cross-sectional survey research study, using a questionnaire with 398 pregnant women | Identify the choice of method of birth and the factors associated with this decision | Most women prefer vaginal birth and this decision is mainly based on the expectation that this method is the most natural and the belief that it is better for the mother-baby bond. |
| Sorrentino  (2022) | Caesarean Section on Maternal Request-Ethical and Juridic Issues: A Narrative Review | Palermo, Italy | Systematic literature review. With article selection criteria | Describe the reasons for the growing demand for cesarean sections chosen by pregnant women | The pregnant woman's autonomy is influenced by negative experiences that lead women to opt for surgical birth because they fear that the fetus will be harmed or because they want a cesarean section for cultural reasons or fear of the unknown. |
| Stoliar  (2023) | A national survey of Australian midwives' birth choices and outcomes | Sydney,  Australia | Prospective observational study using a questionnaire with 447 midwives | Explore the importance of midwives about their experience of giving birth in choosing the method of birth. | Women who are midwives generally have high rates of normal vaginal births and low rates of interventions such as cesarean sections. |
